# An optimized method for purifying, detecting and quantifying *Mycobacterium tuberculosis* RNA from sputum for monitoring treatment response in TB patients

**DOI:** 10.1038/s41598-022-19985-w

**Published:** 2022-10-17

**Authors:** Kayvan Zainabadi, Myung Hee Lee, Kathleen Frances Walsh, Stalz Charles Vilbrun, Laurent Daniel Mathurin, Oksana Ocheretina, Jean William Pape, Daniel W. Fitzgerald

**Affiliations:** 1 Center for Global Health, Weill Cornell Medicine, New York, NY USA; 2 Division of General Internal Medicine, Department of Medicine, Weill Cornell Medicine, New York, NY USA; 3 Les Centres GHESKIO, Port-au-Prince, Haiti

**Keywords:** Microbiology, Applied microbiology, Diagnostic markers

## Abstract

Diagnostics that more accurately detect and quantify viable *Mycobacterium tuberculosis* (Mtb) in the sputum of patients undergoing therapy are needed. Current culture- and molecular-based tests have shown limited efficacy for monitoring treatment response in TB patients, either due to the presence of viable sub-populations of Mtb which fail to grow under standard culture conditions (termed differentially detectable/culturable Mtb, DD Mtb) or the prolonged half-life of Mtb DNA in sputum. Here, we report an optimized RNA-based method for detecting and quantifying viable Mtb from patient sputum during the course of therapy. We first empirically derived a novel RNA extraction protocol from sputum that improves recovery of Mtb RNA while almost completely eliminating contamination from Mtb DNA and host nucleic acids. Next, we identified five Mtb 16S rRNA primer sets with varying limits of detection that were capable of distinguishing between live versus dead H37Rv Mtb. This combined protocol was then tested on sputa from a longitudinal cohort of patients receiving therapy for drug sensitive (DS) or drug resistant (DR) TB with first-line or second-line regimens, respectively. Results were compared with that of culture, including CFU, BACTEC MGIT, and a limiting dilution assay capable of detecting DD Mtb. The five 16S rRNA primer sets positively identified nearly all (range 94–100%) culture positive sputa, and a portion (19–37%) of culture negative sputa. In comparison, ten highly expressed Mtb mRNAs showed positivity in 72–86% of culture positive sputa, and in 0–13% of culture negative sputa. Two of the five 16S rRNA primer sets were able to positively identify 100% of culture positive sputa, and when tested on culture negative sputa from the DS cohort at 2 months post-initiation of therapy, identified 40% of samples as positive; a percentage that is in line with expected treatment failure rates when first-line therapy is discontinued early. These two primer sets also detected 16S rRNA in 13–20% of sputa at 6 months post-initiation of therapy in the DR cohort. Cycle threshold values for 16S rRNA showed a strong correlation with Mtb numbers as determined by culture (R > 0.87), including as Mtb numbers declined during the course of treatment with first-line and second-line regimens. The optimized molecular assay outlined here may have utility for monitoring treatment response in TB patients.

## Introduction

The inability to accurately monitor *Mycobacterium tuberculosis* (Mtb) numbers in the sputa of patients undergoing therapy is compromising patient care and hampering the development of new anti-TB therapeutics. Diagnostics used to assess the early bactericidal activity (EBA) of existing or experimental anti-TB drugs during the first few months of therapy do not correlate well with long-term treatment outcomes^1,2^. An effective diagnostic test of early TB treatment response would allow for identifying patients who may benefit from shorter treatment regimens, and would aid in assessing the efficacy of investigational anti-TB drugs in early phase trials. An assay that accurately detects and quantifies viable Mtb in the sputum of patients undergoing therapy is a good candidate for such a test.

Research indicates that currently available ‘gold standard’ culture-based methods such as colony forming units (CFU) on solid agar or automated liquid culture systems (such as BACTEC MGIT) have limits of detection too high to detect all viable Mtb in clinical samples^1–3^. These methods are known to miss sub-populations of viable but differentially detectable/culturable Mtb (DD Mtb) which may constitute > 90% of the total viable Mtb in sputum, particularly after initiation of first-line therapy^4–8^. These cryptic Mtb populations show profound phenotypic tolerance to anti-TB chemotherapy and thus may have relevance for disease persistence^4,9,10^. Consistent with this notion, studies of TB patients have shown that culture negative lung homogenates are still capable of causing Mtb infection in experimental animals^11^.

Molecular methods for detecting Mtb have shown lower limits of detection than culture, though potentially at the expense of detecting nucleic acids from dead cells^12–14^. An example of this is the Xpert Mtb test which, while highly effective for diagnosing TB infection in treatment naïve individuals, has shown limited efficacy for monitoring treatment response in patients undergoing therapy due to the long half-life of DNA in sputum^12^. Recent studies have confirmed the lack of utility of other DNA-based tests for monitoring response to therapy in TB patients^13–16^.

In comparison to DNA, RNA may serve as a better proxy for viable Mtb owing to its shorter half-life and higher abundance^15–18^. Such an approach has been used to achieve a limit of detection of one pathogen per clinical sample for other infectious diseases^19^. In the case of *M. tuberculosis,* however, the lack of introns in the Mtb genome precludes the use of intron spanning primers to specifically target RNA during RTPCR. Consequently, DNA from non-viable cells can be amplified and lead to a positive test result even in the absence of viable Mtb (i.e. a false positive). Similarly, although more labile than DNA, RNA from dead Mtb cells can also potentially persist and be amplified^14,15^. These concerns are particularly cogent in the case of TB, where dead Mtb cells in the sputum of patients undergoing therapy can outnumber viable cells^3^. The performance of RNA based diagnostics for TB may also be affected by rifamycins, the mainstay of first-line anti-TB treatment regimens, which are known to specifically inhibit Mtb RNA synthesis. New methods which are able to selectively purify and accurately detect and quantitate RNA associated with Mtb viability in sputum are currently needed.

The objective of this study was to develop optimized methods to isolate, detect, and quantitate RNA from viable Mtb in human sputum that could be used to monitor treatment response in TB patients. We first developed a novel RNA isolation protocol from neat sputum that maximized recovery of viable Mtb RNA while minimizing contamination from DNA and other extraneous nucleic acids. We then identified different 16S rRNA primer sets with varying lower limits of detection of Mtb and varying capacity to distinguish between live versus dead laboratory Mtb strain, H37Rv. We tested five of these primer sets, along with ten abundantly expressed Mtb mRNAs, on sputa from TB patients before and during the course of therapy. We studied participants with drug sensitive (DS) TB on rifampin-containing regimens and with drug resistant (DR) TB on non-rifampin containing regimens. We also used a previously described limiting dilution culture assay which allows for enumeration of DD Mtb. The development of this protocol and its performance with respect to culture are discussed below.

## Materials and methods

### Study population and specimen processing

This study was conducted at the Groupe Haïtien d’Étude du Sarcome de Kaposi et des Infectieuses Opportuniste (GHESKIO) center in Haiti, and was approved by both Weill Cornell Medical College and GHESKIO institutional review boards. All participants provided written informed consent and all research was performed in accordance with relevant guidelines and regulations. This study includes sputa from nineteen participants with drug sensitive (DS) and twenty two participants with drug resistant (DR) TB (as determined by Xpert Mtb/RIF test indicative of rifampin resistance) collected between August 2018–January 2021. Detailed information on participant characteristics and inclusion/exclusion criteria have been reported^8^. Participants with DS TB received isoniazid (H), rifampin (R), ethambutol (E), and pyrazinamide (Z) for 2 months, then HR for 4 months. Participants with DR TB received bedaquiline, levofloxacin, linezolid, clofazimine, and pyrazinamide; with bedaquiline discontinued after 6 months and linezolid after 12 months, with the remaining drugs continued to complete 20 months of therapy.

In the current study, we examined overnight sputum collected over the course of 16 h from participants prior to initiation of treatment, and weeks 2, 4 and 8 post-initiation of therapy; with a month 6 collection only performed for subjects with DR TB due to their longer (20 month) treatment regimen. Overnight sputum collection methods have been previously described^7,8^. Some sample collections were missed because of the COVID-19 pandemic, and some sample collections did not yield enough sputum to perform both culture and qRTPCR testing. This resulted in a variable number of sputum samples available at each timepoint from each participant (with the total number of sputum samples available at each timepoint indicated by the n-value in Tables 5, 6). Culture results were kept separate from qRTPCR results to minimize bias. After culture and qRTPCR results were obtained, if a sample showed contamination by all culture methods then it was excluded from the final analysis.

### Microbiological assays

All experiments from here on forth (with the exception of qRTPCR) were performed in a biosafety level 3 laboratory located at GHESKIO and with appropriate safety guidelines and personal protective equipment. Overnight sputum was homogenized by extensive vortexing in a 50 mL falcon tube for 5 min. One portion (5 mL) was decontaminated using NALC-NaOH and used for laboratory cultures including: measure of colony forming units (CFU) on solid agar, BACTEC MGIT, and limiting dilution (LD) assays in liquid media with and without culture filtrate (CF) that is capable of detecting differentially detectable Mtb as described^4,7,8^. Another portion of neat sputum was frozen in 1 mL aliquots at − 80 °C and used for the RNA extraction experiments described below. For purposes of comparing qRTPCR Ct values to Mtb numbers obtained from culture for patient samples, the highest number attained from CFU, LD-CF, or LD+CF was used.

For log-phase H37Rv in vitro studies, only CFU was used for quantitation as a previous study using H37Rv in culture showed comparable numbers obtained with CFU and LD-CF/+CF^4^. For killing studies of H37Rv, rifampin and isoniazid were used at 1 µg/mL, and lack of viability was determined by both CFU and MGIT (H37Rv does not form DD Mtb under these conditions^4^). For HRZE experiments using H37Rv, isoniazid, rifampin, pyrazinamide and ethambutol were used at the following respective concentrations: 0.1 µg/mL, 1 µg/mL, 100 µg/mL and 5 µg/mL. In these experiments, at each timepoint one aliquot of cells was frozen at − 80 °C for RNA while another was washed twice with 7H9 before being plated for CFU determination. RNA was extracted from an equivalent number of Mtb as determined by CFU. RNA extraction from H37Rv involved first spiking into non-Mtb containing sputum (except for the experiment in Table 1). To minimize variation, non-Mtb containing sputa (as determined by negativity by Xpert and Mtb 16S rRNA) were combined, mixed thoroughly, and frozen in large single-use batches.

### Optimization of RNA extraction conditions

**Figure 1:**
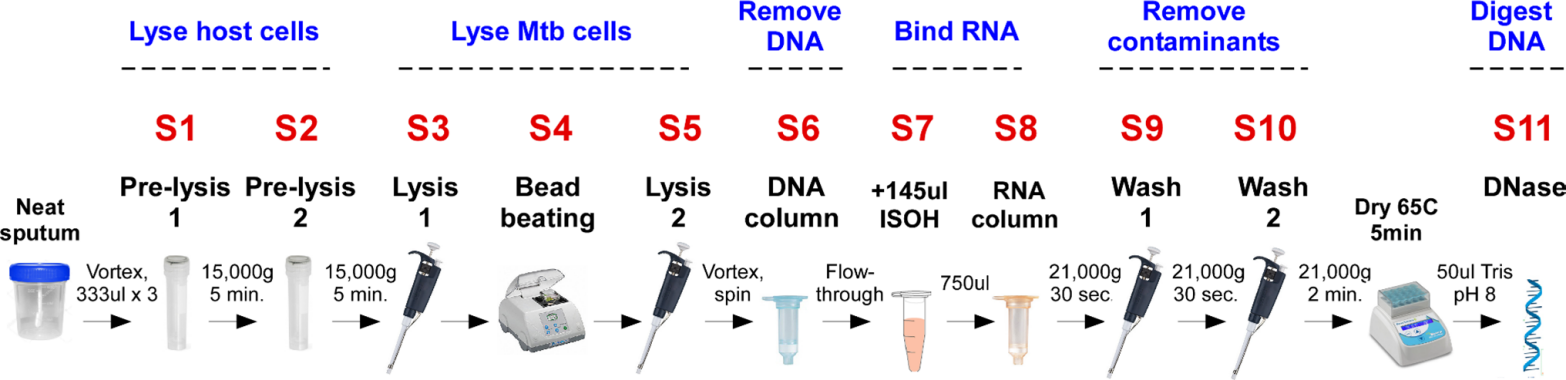
Schematic summary of the Mtb RNA purification protocol from sputum. Numbers on top of each step refer to the Supplemental File containing optimization data for that particular step of the protocol. The final protocol can be found in Tables S14–S16.

Each step of the extraction protocol (Fig. 1) was optimized by monitoring the recovery of Mtb RNA (as measured by qRTPCR for Mtb 16S rRNA, *sigA*, *85b* and/or *icl1*); Mtb DNA (as measured by the same RNA targets but without reverse transcriptase or by the multicopy insertion IS6110 DNA sequence); and host nucleic acids as measured by human *actin* (Tables S1-S11, Figs. S12-S13). Optimization experiments were run using pooled patient sputum samples with at least two replicates and performed at least two independent times.

Sputum samples were extracted and eluted in a final volume of 50 µL of TE buffer pH 8, DNase digested, and 2 µL was used for qRTPCR (in a 10 µL total qRTPCR reaction volume) run typically in triplicate. The final protocols for Mtb RNA (and DNA) extraction, and sources of reagents can be found in Tables S14-S16, S17.

The use of a commercially available internal control (IC) RNA (as part of the QuantiNova Reverse Transcription Kit, Qiagen #205410) was used to assess percent recovery of RNA and to account for presence of qPCR inhibitors. IC was added to the sputa after the second pre-lysis wash (directly into lysis buffer 1) and used for the remainder of the protocol, including bead beating. As a control, IC was added to a sample without sputum; instead 25 µg of carrier DNA (salmon sperm DNA, Sigma #262012), 25 µg of carrier RNA (RNA from yeast, Sigma #10109223001), and 50 µg of BSA (New England Biolabs #B9000S) were added. IC was amplified with the QuantiNova IC Probe Assay (Qiagen #205813).

### qPCR conditions

Quantitative PCR for *M. tuberculosis* 16S rRNA, mRNA targets, and human *actin* was performed as a multiplexed reaction (incorporating a maximum of three primer sets) using QuantiTect multiplex RTPCR master mix (Qiagen #204645) and a Roche LC96 instrument. The use of the Qiagen QuantiTect multiplex kit is advised, as we have found it to be highly resistant to qRTPCR inhibitors^20–22^. In the case of reverse-transcription (RT) PCR, Qiagen RT-MIX was added to the master mix and a RT step consisting of 50 °C for 20 min was included (Table S18). DNA-based qPCR followed similar cycling conditions, except the RT enzyme was left out of the reaction. To increase sensitivity for detecting Mtb DNA and human DNA/RNA contamination, the smallest amplicon size was chosen for the Mtb multicopy IS6110 DNA marker (71 bp) and the human *actin* gene (60 bp); larger amplicon sizes for both often failed to produce Ct values ≤ 35 during optimization experiments.

Sequences for 16S rRNA primers ‘F1 R1’ and probe were obtained from Choi et al.^23^, which have previously been shown to be specific to Mtb. We confirmed this by digital PCR (which indicated the probe was highly specific for Mtb) and by testing on 10 non-TB sputum samples. While keeping the probe constant, additional primers were developed to yield varying amplicon sizes, and tested at two different primer concentrations (0.1 µM and 0.5 µM). All primer and probe sequences were purchased from Thermofisher, except for cy5 probes which were purchased from TIB-Molbiol. Primer and probe sequences and cycling conditions are presented in Table S18.

For mRNA targets, qRTPCR expression data from Walter et al.^24^ was used to identify ten highly expressed mRNAs from TB patient sputum samples. For each gene, at least four different primers (and one probe) were designed in order to yield a total of four possible amplicons. These were then tested on RNA extracted from pooled pre-treatment TB patient sputum samples to identify the primer set that provided the lowest Ct value, which was then used for subsequent experiments.

In order for a sample to be considered positive, all qRTPCR replicates had to be positive and all qPCR replicates without RT had to be negative. In cases of ambiguous results, the sample was re-run by qRTPCR and whenever possible, RNA was re-extracted. Maximum cycle threshold (MCT) and minimal endpoint fluorescence (EPF) values were determined empirically by testing serial dilutions of log-phase H37Rv, and H37Rv killed with rifampin and isoniazid for four weeks. Absolute numbers for MCT and EPT varied occasionally with each new batch of Qiagen QuantiTect multiplex RTPCR master mix, though MCT values were typically 34–35 and EPF values 0.1–0.15.

Of note, in the present report we avoid using the terms “sensitivity” and “specificity” because we recognize that current gold standard culture techniques do not detect all viable Mtb. We instead use the terms “lower limit of detection (LoD)” and “ability to distinguish between live/dead H37Rv” in lieu of sensitivity and specificity, respectively.

## Results

### Developing a novel Mtb RNA-specific purification method from sputum

In order for RNA to serve as an accurate marker of Mtb viability it is important that nucleic acids from non-viable sources be absent. To achieve this, a novel method for purifying Mtb RNA from sputum was devised. Neat sputum was chosen as the starting material since decontamination has been shown to negatively impact Mtb RNA recovery^25^. Each step of the protocol was optimized by identifying conditions that improved recovery of Mtb RNA, but not Mtb DNA or host nucleic acids, based on qPCR cycle threshold (Ct) values (Fig. 1, Tables S1–S11). To reduce the amount of nucleic acids originating from dead Mtb cells, sputum samples were washed with two different buffers prior to RNA extraction (first an anionic detergent containing 2-mercaptoethanol, followed by a guanidine thiocyanate solution lacking detergent). These washes also helped to lyse most non-Mtb cells (without lysing Mtb itself) and served as effective mucolytic agents, improving bead beating efficiency and allowing for higher volumes of sputum (≥ 1 mL) to be utilized (Tables S1–S2).

Given the importance of preventing DNA contamination, two independent methods for eliminating DNA were employed. First, lysates are passed through a commercial DNA column where DNA is bound and thereby removed (with the resulting flow-through lysate used for RNA extraction) (Table 1, Table S6). Second, the final purified RNA is treated with DNase to digest away any remaining DNA (Table S11). The combination of these two steps proved essential to ensure that DNA is consistently kept at undetectable levels when tested with single-copy Mtb genes (and at undetectable or near undetectable levels when tested with the more sensitive multicopy IS6110 DNA marker) (Table 2). A tangential benefit of removing DNA and host nucleic acids prior to RNA extraction is that recovery of Mtb RNA is markedly improved (Table 2, Tables S1–S2). The final protocol requires approximately two hours from start to finish and is compatible with basic laboratory equipment and reagents.

**Table 1:** Passing Mtb lysates through a DNA column prior to RNA purification captures a substantial portion of the Mtb DNA.

	H37Rv			Sputum (Day 0)			Sputum (Week 2 PI)		
	Total input	DNA eluate	RNA eluate	Total input	DNA eluate	RNA eluate	Total input	DNA eluate	RNA eluate
16S rRNA Avg Ct ± SD	8.4 ± 0.5	13.4 ± 0.3	8.6 ± 0.2	16.8 ± 0.5	18.2 ± 0.1	15.2 ± 0.1	21.0 ± 0.3	21.0 ± 0.2	27.7 ± 0.2
IS6110 DNA Avg Ct ± SD	9.6 ± 0.1	11.1 ± 0.2	22.7 ± 0.2	17.0 ± 0.1	17.3 ± 0.1	28.3 ± 0.1	18.8 ± 0.2	18.7 ± 0.4	33.5 ± 1.5

Total input represents the nucleic acids recovered from the RNA column when the Mtb lysate was not first passed through a DNA column to remove DNA; DNA eluate represents nucleic acids recovered from the DNA column after the Mtb lysate was passed through it; and RNA eluate represents nucleic acids recovered from the RNA column when the Mtb lysate was first passed through the DNA column to remove DNA.

Samples were not DNase treated; Average cycle threshold values from three replicates for each condition are presented, *PI* post-initiation of therapy with first-line drugs, *Avg* average, *SD* standard deviation.

**Table 2:** The combination of a two-step DNA removal process (DNA column and DNase digestion) removes more Mtb DNA than either alone.

Step 1	− DNA column step		+ DNA column step	
Step 2	− DNase	+ DNase	− DNase	+ DNase
16S rRNA Avg Ct ± SD	18.7 ± 1.7	18.8 ± 0.1	14.0 ± 0.2	14.9 ± 1.2
IS6110 DNA Avg Ct ± SD	15.8 ± 0.8	29.9 ± 0.2	28.8 ± 0.2	Undetectable

A secondary benefit of DNA removal prior to RNA purification is improved recovery of Mtb 16S rRNA.

Average cycle threshold values from three replicates for each condition using a patient sputum sample are presented; Avg, average; SD, standard deviation.

### Comparison to existing sputum-based RNA purification methods

**Figure 2:**
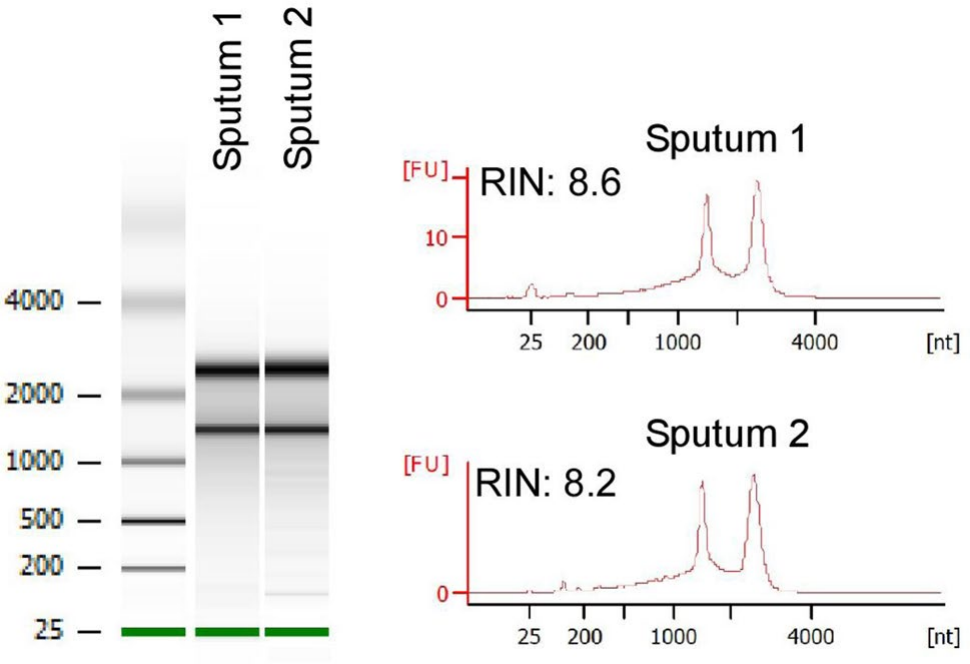
Agilent Bioanalyzer results for RNA extracted with the current protocol for two TB patient sputum samples demonstrating lack of genomic DNA and eukaryotic 18S rRNA contamination, and RNA integrity numbers (RIN) of  > 8.

To determine how the final protocol fared in comparison to existing methods, RNA from three TB patient sputum samples was extracted with this and two previously published Mtb RNA extraction methods^15,18,26,27^. For all three samples, the current protocol purified more Mtb RNA (as measured by 16S rRNA and *icl1* mRNA), less Mtb DNA (as measured by the ratio of Mtb IS6110 DNA/16S rRNA) and markedly less host RNA (as measured by human *actin*) (Table 3). Further, no evidence of qRTPCR inhibition was observed when an internal control RNA was included. Agilent Bioanalyzer analysis confirmed these results: RNA extracts obtained with the current protocol were free of DNA and eukaryotic 18S rRNA contamination, and had RNA integrity numbers > 8 (Fig. 2).

**Table 3:** Performance of the current RNA extraction protocol in comparison to two previously published methods for purification of Mtb RNA using three TB patient sputum samples.

	Sputum A (3.3 × 10^6^ CFU/mL)					Sputum B (7.0 × 10^6^ CFU/mL)					Sputum C (3.8 × 10^6^ CFU/mL)				
	16S rRNA^1^	icl1 mRNA	IS6110 DNA^3^	Human actin	IC RNA^2^	16S rRNA^1^	icl1 mRNA	IS6110 DNA^3^	Human actin	IC RNA^2^	16S rRNA^1^	icl1 mRNA	IS6110 DNA^3^	Human actin	IC RNA^2^
Current study	12.2 ± 0.1	21.2 ± 0.2	33.0 ± 0.7	33.1 ± 0.7	28.2 ± 0.1	**11.8 ± 0.1**	**19.4 ± 0.3**	**32.0 ± 0.7**	**33.5 ± 0.8**	**28.5 ± 0.2**	13.9 ± 0.1	23.0 ± 0.2	32.8 ± 0.3	34.8 ± 2.0	28.5 ± 0.1
Honeyborne et al.^18^	15.9 ± 0.1	26.4 ± 0.1	32.0 ± 0.7	24.5 ± 0.1	29.1 ± 0.1	**14.0 ± 0.1**	**22.2 ± 0.1**	**30.3 ± 0.1**	**22.1 ± 0.1**	**28.3 ± 0.3**	15.0 ± 0.1	24.9 ± 0.1	29.7 ± 0.3	23.7 ± 0.1	29.3 ± 0.1
Desjardin et al.^15^	16.0 ± 0.1	25.5 ± 0.1	36.0 ± 0.8	18.0 ± 0.1	28.8 ± 0.1	**15.7 ± 0.1**	**23.5 ± 0.1**	**37.6 ± 0.1**	**18.4 ± 0.1**	**28.5 ± 0.1**	16.7 ± 0.3	26.4 ± 0.3	37.1 ± 0.9	19.2 ± 0.2	28.6 ± 0.1

Average cycle threshold values from three replicates are presented for Mtb RNA targets (16S rRNA and icl1 mRNA), one Mtb DNA target (multicopy IS6110), one host target (human actin), and an internal control RNA (IC RNA) which was used to assess percent recovery of RNA and/or presence of qPCR inhibitors.

^1^Amplified with primer set F1 R1 (0.1 µM).

^2^Extraction of internal control (IC) RNA without sputum yielded a Ct value of 28.2 ± 0.1 (see “Materials and methods” section).

^3^DNase digestion was performed equivalently for all three methods (15 min at 37 °C); CFU, colony forming units.

Sputum B columns are bolded for visual aid.

### Developing an optimized qRTPCR detection strategy for Mtb

Owing to its higher abundance and longer half-life than mRNA (but shorter half-life than DNA), Mtb 16S rRNA makes an attractive target for qRTPCR. However, its longer half-life posed a potential problem—unless 16S rRNA from non-viable sources degrades beyond the qRTPCR amplicon size then it may still be detectable and result in false positive results^12,16^. We therefore sought to develop an optimized qRTPCR detection strategy that was demonstrably able to distinguish between viable versus non-viable Mtb. We used the published primer/probe set from Choi et al.^23^ to design five additional primers (while keeping the Mtb-specific probe constant) to yield 16S rRNA amplicons of varying sizes (77–146 bp).

These were then tested at two different concentrations (0.1 µM and 0.5 µM) on serial dilutions of laboratory Mtb strain, H37Rv (spiked into non-Mtb containing sputum) to gauge their approximate limits of detection (LoD); and on a sterilized culture of H37Rv (killed with rifampin and isoniazid for four weeks) to measure their ability to distinguish between live versus dead Mtb. As can be seen in Table 4, decreasing the amplicon size, or increasing the primer concentration, generally improved the LoD but worsened the ability of the primer sets to distinguish between live/dead H37Rv. In fact, a clear inverse trend emerged: primer sets with LoDs of  ≤ 10^3^ H37Rv/mL were unable to distinguish between live/dead H37Rv, whereas primers with LoDs of  > 10^3^ H37Rv/mL were able to do so. Notably, standard curves for all 16S rRNA primer sets showed a strong correlation between qRTPCR cycle threshold (Ct) values and log-phase H37Rv cell numbers as measured by CFU (median R = 0.98) (Fig. S12).

**Table 4:** Average qRTPCR cycle threshold values for eight different 16S rRNA qRTPCR reaction conditions (consisting of various primer combinations and concentrations) tested on serial dilutions of H37Rv spiked into non-Mtb containing sputum (top), or log-phase H37Rv killed by rifampin (RIF) and isoniazid (INH) over the course of 4 weeks (bottom).

	Primer set:	F1 R1*^1^	F2 R1*	F1 R1*^1^	F2 R1*	F1.2 R1*	F1.1 R1.2	F1.2 R1.2	F1.2 R4*^4^
	Amplicon size:	146	92	146	92	95	81	77	80
	Primer conc:	0.1 µM	0.1 µM	0.5 µM	0.5 µM	0.5 µM	0.5 µM	0.5 µM	0.5 µM
Limit of detection (LoD) of H37Rv	0 CFU/mL	–	–	–	–	–	–	–	–
	10^1^ CFU/mL	–	–	–	–	–	–	–	30.4
	10^2^ CFU/mL	–	–	–	–	–	–	28.3	28.6
	10^3^ CFU/mL	–	–	–	–	–	26.3	25.0	25.1
	10^4^ CFU/mL	–	–	–	21.0	20.7	21.2	21.5	21.6
	10^5^ CFU/mL	–	17.5	16.8	16.1	16.1	17.8	18.1	18.2
	10^6^ CFU/mL	14.0	13.8	13.0	12.8	12.9	14.7	14.7	14.8
	10^7^ CFU/mL	10.9	10.5	10.3	9.9	10.1	11.7	11.5	11.5
Ability to distinguish live/dead H37Rv	RIF + INH Wk0: (5 × 10^6^ CFU/mL)	12.2	12.2	11.3	11.4	11.2	12.8	13.5	12.1
	RIF + INH Wk1: (1.3 × 10^3^ CFU/mL)	17.3	16.2	15.5	14.9	14.4	15.9	16.9	15.7
	RIF + INH Wk2: (1 × 10^1^ CFU/mL)	–	–	–	19.5	19.0	19.9	21.0	19.4
	RIF + INH Wk4: (0 CFU/mL)^2,3^	–	–	–	–	–	22.2	23.1	22.8

The first five primer sets with LoDs of  ≥ 10^4^ H37Rv/mL are able to distinguish live from dead H37Rv, whereas the remaining primer sets with lower LoDs are not. Primers sets chosen for subsequent experiments are indicated by *.

*Wk* week, *conc* concentration, *CFU* colony forming unit, *Mtb Mycobacterium tuberculosis*.

^1^Original 16S rRNA primer set from Choi et al.^23^.

^2^Also negative by BACTEC MGIT.

^3^H37Rv does not form DD Mtb under these conditions^4^.

^4^While unable to distinguish between live/dead H37Rv, this primer was included in subsequent analyses as a comparator since it has the lowest LoD of any primer set tested.

### Testing 16S rRNA primers on TB patient sputa samples

We next chose the five primer sets that were capable of distinguishing between live/dead H37Rv (as well as one which could not but which had the lowest LoD as a comparator) for further evaluation. Since it was unclear how well the above in vitro experiments corresponded to actual TB patient sputa, we first compared the H37Rv standard curves to ones obtained with serial dilutions of a TB patient sputum sample. As can be seen in Fig. S12, standard curves between the two matched closely.

**Figure 3:**
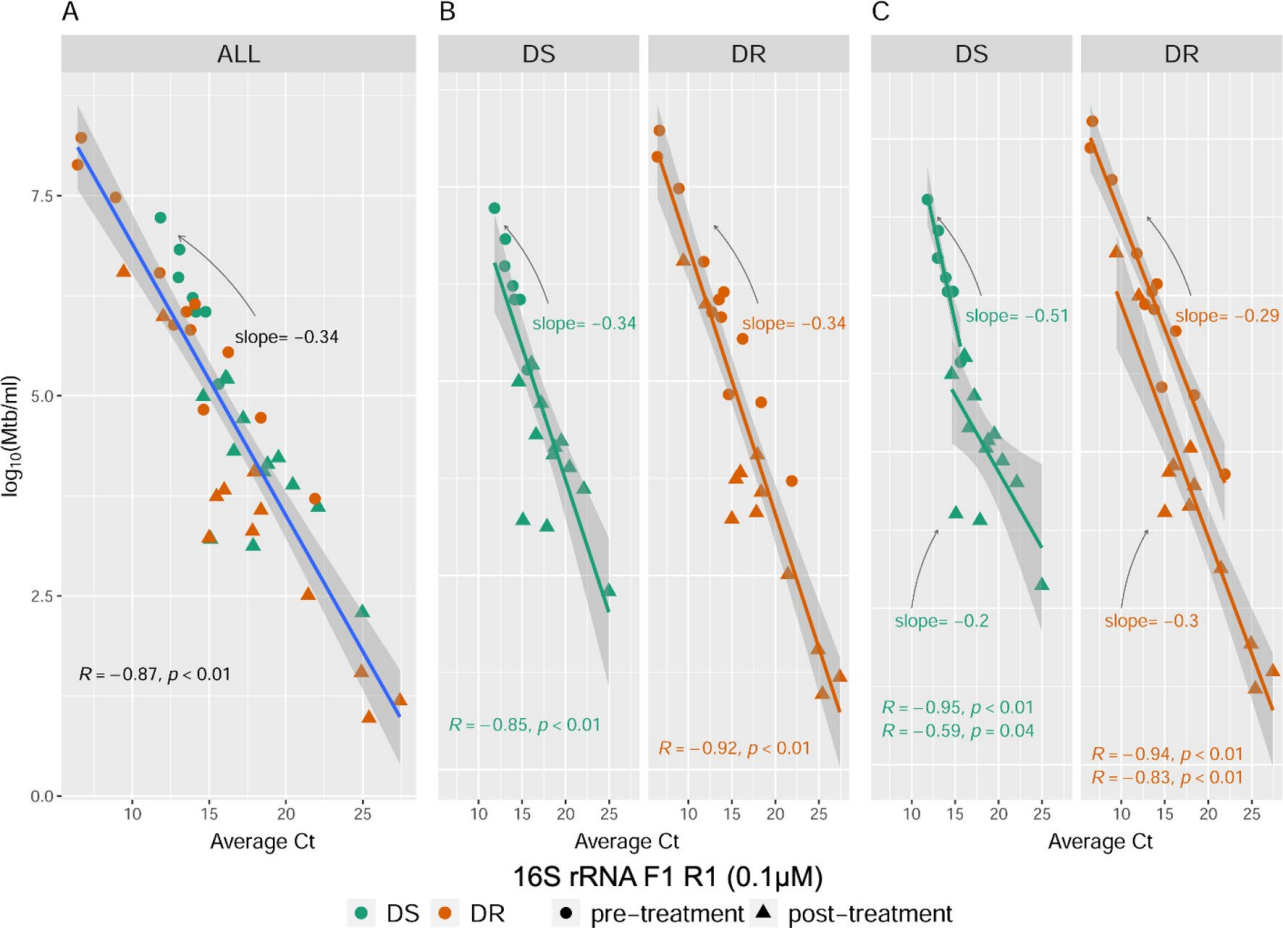
qRTPCR cycle threshold values for a representative 16S rRNA primer set (F1 R1 0.1 µM) show a strong correlation with *M. tuberculosis* (Mtb) numbers from patient sputum samples when analyzing all samples (both pre- and post-initiation of treatment) from both cohorts (**A**); or each cohort individually (**B**). This correlation decreases for subjects with drug sensitive TB (DS) after initiation of a rifampin-based combination therapy (**C**). Mtb numbers represent the highest Mtb/mL obtained by either CFU or limiting dilution assays with or without culture filtrate. Results for the other 16S rRNA primer sets are presented in Fig. S13.

**Figure 4:**
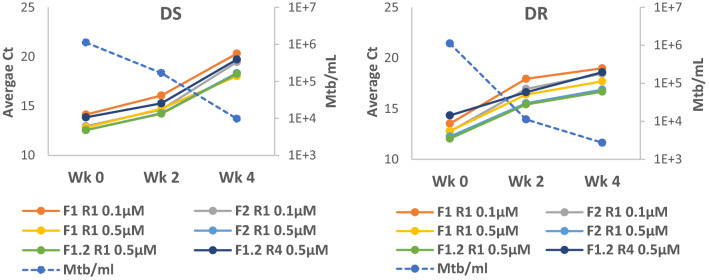
Average cycle threshold values for the different 16S rRNA primer sets (left axis) and Mtb counts (right axis) for sputa from a subject with drug sensitive (DS) or drug resistant (DR) TB during the course of therapy with first-line or second-line drug regimens, respectively. Mtb numbers (shown by dashed line) represent the highest number obtained by either CFU or limiting dilution assays with or without culture filtrate. The final primer set ‘F1.2 R4 0.5 µM’ (dark blue solid line) was unable to distinguish between live/dead H37Rv (Table 4) but was included as a comparator since it has the lowest limit of detection of any 16S rRNA primer set tested.

We next tested the 16S rRNA primer sets on sputa obtained from subjects with drug sensitive (DS) or drug resistant (DR) TB—herein referred to as the DS and DR cohorts—before and after initiation of first-line (rifampin based) or second-line (non-rifampin based) therapies, respectively (Table S19). Overall, qRTPCR Ct values showed a strong correlation with Mtb numbers as determined by culture when comparing all samples (both pre- and post-initiation of therapy sputa from both cohorts; median R = 0.88), or when analyzing each cohort individually (median R for the DS and DR cohorts = 0.91 and 0.92, respectively) (Fig. 3, Fig. S13). This correlation decreased for the DS cohort after initiation of a rifampin-based treatment regimen (median pre- vs post-treatment R = 0.97 vs 0.72, respectively), but less so for the DR cohort who do not take rifampin (median pre- vs post-treatment R = 0.95 vs 0.87, respectively) (Fig. 3, Fig. S13). Importantly, declining Mtb numbers during the course of therapy for subjects from the DS and DR cohorts corresponded with increasing qRTPCR Ct values for 16S rRNA (Fig. 4). These experiments also revealed that the LoDs for the 16S rRNA primers were in fact lower than initially suggested by the H37Rv experiments: < 10^2^ Mtb/mL for the first three primers which previously showed LoDs of  ≥10^5^ with H37Rv/mL; and < 10^1^ Mtb/mL for the rest.

### Performance of 16S rRNA qRTPCR in comparison to culture for positive detection of Mtb in sputum

The performance of the 16S rRNA primer sets was next compared to culture as a binary outcome (positive versus negative) for detection of Mtb from patient sputa. Prior to initiation of therapy, all five 16S rRNA primer sets positively identified 100% of culture positive sputa (defined as positive by either CFU, BACTEC MGIT, or a limiting dilution assay capable of detecting differentially detectable Mtb) from both the DS and DR cohorts (Table 5, Table S19)^4,7,8^.

**Table 5:** qRTPCR positivity by the different 16S rRNA primer sets in comparison to culture for sputa from subjects with drug sensitive (DS) or drug resistant (DR) TB receiving first-line or second-line therapies, respectively.

Culture status:	Day 0				Week 2				Month 1				Month 2				Month 6^1^	
	DS (n = 7)		DR (n = 12)		DS (n = 8)		DR (n = 12)		DS (n = 12)		DR (n = 4)		DS (n = 12)		DR (n = 17)		DR (n = 15)	
	7+	0−	12+	0−	8+	0−	12+	0−	10+	2−	3+	1−	2+	10−	4+	13−	0+	15−
F1 R1 (0.1 µM)+	7	0	12	0	7	0	12	0	8	0	3	0	1	1	4	6	0	1
F1 R1 (0.1 µM)−	0	0	0	0	1	0	0	0	2	2	0	1	1	9	0	7	0	14
**F2 R1 (0.1 µM)+**	**7**	**0**	**12**	**0**	**8**	**0**	**12**	**0**	**8**	**0**	**3**	**0**	**1**	**2**	**4**	**7**	**0**	**1**
**F2 R1 (0.1 µM)−**	**0**	**0**	**0**	**0**	**0**	**0**	**0**	**0**	**2**	**2**	**0**	**1**	**1**	**8**	**0**	**6**	**0**	**14**
F1 R1 (0.5 µM)+	7	0	12	0	8	0	12	0	8	0	3	1	1	2	4	7	0	1
F1 R1 (0.5 µM)−	0	0	0	0	0	0	0	0	2	2	0	0	1	8	0	6	0	14
**F2 R1 (0.5 µM)+**	**7**	**0**	**12**	**0**	**8**	**0**	**12**	**0**	**10**	**0**	**3**	**1**	**2**	**4**	**4**	**7**	**0**	**2**
**F2 R1 (0.5 µM)− **	**0**	**0**	**0**	**0**	**0**	**0**	**0**	**0**	**0**	**2**	**0**	**0**	**0**	**6**	**0**	**6**	**0**	**13**
F1.2 R1 (0.5 µM)+	7	0	12	0	8	0	12	0	10	0	3	1	2	4	4	7	0	3
F1.2 R1 (0.5 µM)−	0	0	0	0	0	0	0	0	0	2	0	0	0	6	0	6	0	12
**F1.2 R4 (0.5 µM)+**^**2**^	**7**	**0**	**12**	**0**	**8**	**0**	**12**	**0**	**10**	**0**	**3**	**1**	**2**	**7**	**4**	**8**	**0**	**5**
**F1.2 R4 (0.5 µM)−**	**0**	**0**	**0**	**0**	**0**	**0**	**0**	**0**	**0**	**2**	**0**	**0**	**0**	**3**	**0**	**5**	**0**	**10**

^1^Month 6 post-initiation of therapy samples were only collected from subjects with DR TB due to their longer (20 month) treatment regimen.

^2^The final primer set ‘F1.2 R4 0.5 µM’ was unable to distinguish between live/dead H37Rv (Table 4) but was included as a comparator since it has the lowest limit of detection of any 16S rRNA primer set tested; n-values represent number of sputum available at each timepoint for each cohort (see “Materials and methods” section); Cycle threshold (Ct) values can be found in Table S19.

Rows corresponding to different primer sets are alternately bolded for visual aid.

After initiation of therapy, the 16S rRNA primer sets began to show differences, both with respect to one another and between the DS and DR cohorts (Table 5, Table S19). For instance, all five 16S rRNA primer sets were able to positively identify 100% of culture positive post-initiation of treatment sputa from the DR, but not DS cohort. In the DS cohort, the three primer sets with the higher LoDs (< 10^2^ Mtb/mL) identified 90–92% of culture positive sputa, whereas the two primer sets with the lower LoDs (< 10^1^ Mtb/mL) identified 100% of culture positive sputa.

**Figure 5:**
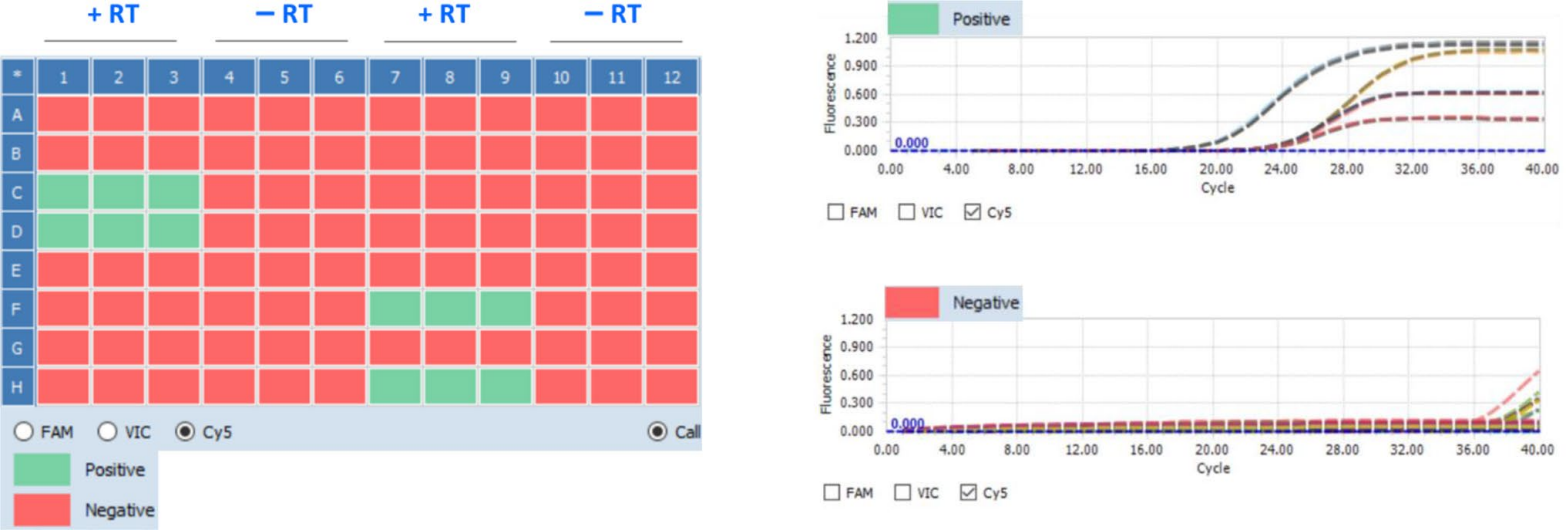
16S rRNA qRTPCR results for month two post-initiation of therapy sputa from the DS and DR cohorts performed with and without reverse transcriptase (RT) confirms RNA-specific amplification. qRTPCR results for sixteen sputa samples amplified with primer set 16S rRNA F1 R1 (0.1 µM), each performed in triplicate, are presented. Lack of amplification when reverse transcriptase (RT) is left out of the reaction (left panel) confirms lack of Mtb DNA contamination. Amplification curves for positive and negative samples are shown on the right. For completeness, amplification curves for all negative samples (both with and without reverse transcriptase) are shown (bottom right panel). Each group of three boxes (such as C1–C3) represents one sputum sample performed in triplicate either with (+ RT) or without RT (− RT).

We next ascertained whether the five 16S rRNA primer sets were able to detect 16S rRNA in patient sputa that were culture negative (defined as negative by all culture methods). Over all timepoints, the positivity rates for the five 16S rRNA primer sets were 8–33% for the DS cohort and 24–38% for the DR cohort in culture negative sputa (Table 5, Table S19). At month two specifically, the positivity rates for the five primer sets were 10–40% for the DS cohort and 46–54% for the DR cohort; and at month six it was 7–20% for the DR cohort (who adhere to a 20 month treatment regimen). Of note, in these experiments 16S rRNA amplification was absent when reverse transcriptase was left out of the qRTPCR reaction, confirming RNA specific amplification (Fig. 5).

Of the five 16S rRNA primer sets capable of differentiating between live/dead H37Rv, only two (‘F2 R1’ and ‘F1.2 R1’ both at 0.5 µM) were able to identify 100% of culture positive sputa from the DS and DR cohorts. When limiting analyses to culture negative sputa positive by both of these primer sets, the qRTPCR positivity rate at month two was 40% for the DS cohort and 54% for the DR cohort; and 13% for the DR cohort at month six. When using the strictest measure—sputa positive by all five 16S rRNA primer sets—the qRTPCR positivity rate for culture negative sputa at month two falls to 10% for the DS cohort and 46% for the DR cohort; and to 7% at month six for the DR cohort.

### Comparison of 16S rRNA to mRNA targets for qRTPCR

Given its shorter half-life (and lower abundance), we next examined how Mtb mRNA compared to 16S rRNA as targets for qRTPCR. We chose ten abundantly expressed Mtb mRNAs based on the work of Walter et al.^24^ (median day 0 expression, > 98th percentile) and compared positivity rates to 16S rRNA in a subset of DS and DR patient sputa. Prior to initiation of therapy, the ten mRNA targets performed nearly as well as 16S rRNA for positively identifying culture positive sputa (range of 84–100%) (Table 6). Concordance with culture positivity, however, fell after initiation of therapy: the ten mRNA targets positively identified 42–74% of culture positive sputa from the DS cohort and 63–89% of culture sputa from the DR cohort (Table 6). When tested on culture negative sputa, only three of the ten mRNA targets showed any positivity: *hspX* was the sole gene in the DS cohort to show positivity at month two (10%) (Table S19); and *arsC* and *tatA* were the sole genes in the DR cohort to show positivity at month two (14% and 29%, respectively) (Table 6). No sputa tested positive for any mRNAs at month six in the DR cohort, and all sputa positive by mRNA were also positive for 16S rRNA (Table S19).

**Table 6:** qRTPCR positivity by ten abundantly expressed Mtb mRNA targets in comparison to culture for sputa from subjects with drug sensitive (DS) or drug resistant (DR) TB receiving first-line or second-line therapies, respectively.

Culture status:	Day 0				Week 2				Month 1				Month 2			
	DS (n = 7)		DR (n = 12)		DS (n = 8)		DR (n = 12)		DS (n = 11)		DR (n = 4)		DS (n = 7)		DR (n = 11)	
	7+	0−	12+	0−	8+	0−	12+	0−	10+	1−	3+	1−	1+	6−	4+	7−
hspX+	7	0	12	0	5	0	9	0	8	0	3	0	0	1	3	0
hspX−	0	0	0	0	3	0	3	0	2	1	0	1	1	5	1	7
**vapB10+**	**7**	**0**	**12**	**0**	**7**	**0**	**10**	**0**	**7**	**0**	**2**	**0**	**0**	**0**	**4**	**0**
**vapB10−**	**0**	**0**	**0**	**0**	**1**	**0**	**2**	**0**	**3**	**1**	**1**	**1**	**1**	**6**	**0**	**7**
icl1+	7	0	12	0	5	0	10	0	7	0	3	0	0	0	4	0
icl1−	0	0	0	0	3	0	2	0	3	1	0	1	1	6	0	7
**lldD2+**	**7**	**0**	**12**	**0**	**5**	**0**	**8**	**0**	**7**	**0**	**2**	**0**	**0**	**0**	**2**	**0**
**lldD2−**	**0**	**0**	**0**	**0**	**3**	**0**	**4**	**0**	**3**	**1**	**1**	**1**	**1**	**6**	**2**	**7**
carD+	7	0	12	0	6	0	9	0	7	0	3	0	0	0	4	0
carD−	0	0	0	0	2	0	3	0	3	1	0	1	1	6	0	7
**whiB1+**	**7**	**0**	**12**	**0**	**5**	**0**	**9**	**0**	**7**	**0**	**2**	**0**	**0**	**0**	**4**	**0**
**whiB1−**	**0**	**0**	**0**	**0**	**3**	**0**	**3**	**0**	**3**	**1**	**1**	**1**	**1**	**6**	**0**	**7**
tatA+	7	0	12	0	3	0	8	0	8	0	3	0	0	0	3	2
tatA−	0	0	0	0	5	0	4	0	2	1	0	1	1	6	1	5
**Rv1738+**	**7**	**0**	**11**	**0**	**3**	**0**	**9**	**0**	**5**	**0**	**3**	**0**	**0**	**0**	**3**	**0**
**Rv1738−**	**0**	**0**	**1**	**0**	**5**	**0**	**3**	**0**	**5**	**1**	**0**	**1**	**1**	6	**1**	**7**
arsC+	7	0	11	0	5	0	10	0	7	0	3	0	0	0	4	1
arsC−	0	0	1	0	3	0	2	0	3	1	0	1	1	6	0	6
**lpqX+**	**7**	**0**	**9**	**0**	**5**	**0**	**9**	**0**	**6**	**0**	**2**	**0**	**0**	**0**	**3**	**0**
**lpqX−**	**0**	**0**	**3**	**0**	**3**	**0**	**3**	**0**	**4**	**1**	**1**	**1**	**1**	**6**	**1**	**7**

n-values represent number of sputum available at each timepoint for each cohort (see “Materials and methods” section); Cycle threshold (Ct) values can be found in Table S19.

Rows corresponding to different mRNA targets are alternately bolded for visual aid.

### Rifampin decreases 16S rRNA and mRNA abundance

The 16S rRNA and mRNA targets showed lower positivity rates for culture positive sputa from the DS than DR cohort after, but not before, initiation of therapy. This is in spite of the fact that at week two post-initiation of therapy, the median Mtb/mL was actually 2.6-fold higher for sputa from the DS than DR cohort. We hypothesized this difference may be attributable to rifampin, an Mtb RNA polymerase inhibitor which is part of the drug regimen taken by DS, but not DR subjects.

**Figure 6:**
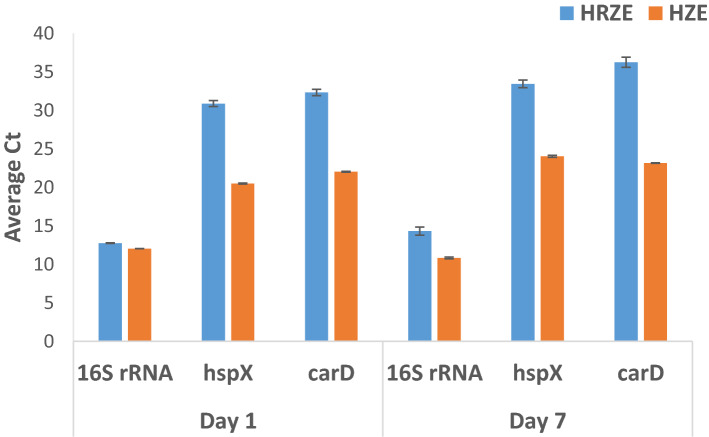
Average qRTPCR cycle threshold values for 16S rRNA, and hspX and carD mRNAs for H37Rv cells treated with isoniazid, pyrazinamide and ethambutol either with rifampin (HRZE) or without rifampin (HZE). RNA was extracted from an equivalent number of cells based on CFU after treatment with HRZE or HZE. Analyses were performed at day 1 and day 7 after addition of drugs. Results for 16S rRNA primer set ‘F1 R1 0.1 µM’ are shown.

To test this, we treated log-phase H37Rv with isoniazid, pyrazinamide and ethambutol either with rifampin (HRZE) or without rifampin (HZE) and assessed 16S rRNA and mRNA levels by qRTPCR (taking care to extract from an equivalent number of cells at each timepoint based on CFU). In comparison to HZE treatment**,** mRNA levels declined markedly after 1 day of HRZE treatment and remained that way at day 7 (Fig. 6). 16S rRNA levels showed a more time-dependent decline with HRZE treatment, with modest effects at day 1 which became more prominent at day 7, likely reflecting the longer half-life of 16S rRNA.

## Discussion

The objective of this study was to develop optimized methods for the isolation, detection, and quantification of RNA associated with viable Mtb in the sputa of patients undergoing anti-tuberculous therapy. To achieve this we first empirically derived a novel RNA extraction method from sputum that improved recovery of Mtb RNA compared to previously published methods and which almost completely eliminated contamination from Mtb DNA and host nucleic acids. We then developed a panel of Mtb 16S rRNA primer sets consisting of varying amplicon sizes and primer concentrations. In vitro, this panel had varying lower limits of detection of viable H37Rv Mtb, which was inversely related to their ability to distinguish live from dead Mtb. Testing this panel on sputum of TB patients undergoing therapy gave rise to varying positivity rates in culture positive and culture negative sputa, consistent with in vitro studies. Further, the quantity of 16S rRNA as measured by qRTPCR Ct value correlated well with the quantity of Mtb measured by culture, including an assay for differentially detectable Mtb. Large clinical studies are needed to correlate detection and quantity of Mtb RNA in sputum with TB treatment outcomes.

The persistence of Mtb DNA in sputum after cell death and the difficulty of specifically targeting Mtb RNA during RTPCR necessitated the development of a new RNA purification protocol. To reduce contamination by Mtb DNA, two independent methods for removing DNA were utilized; and to reduce the amount of nucleic acids originating from dead Mtb cells, sputa samples were washed with two different buffers prior to RNA extraction. These precautions were taken in order to increase the likelihood that detection of Mtb RNA in sputum correlated with Mtb viability. These steps also helped to remove most non-Mtb nucleic acids prior to RNA extraction, thereby enriching for, and improving the recovery of, Mtb RNA—a quality that may have applicability for other RNA-based studies from sputum. For instance, the ability to eliminate most host nucleic acids prior to RNA extraction may have relevance for RNA sequencing studies from TB patient sputa.

We also systematically evaluated and identified five 16S rRNA primer sets that were capable of distinguishing between live versus dead H37Rv. Three of these primer sets showed positivity in only 90–92% of culture positive sputa from the DS cohort after initiation of therapy, yet still showed positivity for 16S rRNA in 10–20% of culture negative sputa from the same cohort after two months of therapy. In the DR cohort, a similar percentage (7–20%) of culture negative sputa at month six tested positive for 16S rRNA by at least one of the five primer sets. Positivity for 16S rRNA late during treatment (months 2–6) may predict patients at greater risk for treatment failure or recurrence. Conversely, negativity by both culture and 16S rRNA may identify patients who are responding well to therapy and thus may qualify for shorter treatment regimens. Of note, 60% of culture negative sputa from the DS cohort at month two were also negative by all five 16S rRNA primer sets; a percentage that is in line with expected treatment success rates when first-line therapy is discontinued early (< 4 months)^28–30^. A longitudinal study with a large TB patient population is needed to determine whether RNA positivity at months 2–6 does in fact correlate with treatment outcomes.

In comparison to 16S rRNA, ten highly expressed Mtb mRNAs, including ones used in previous studies^17,31^, showed lower positivity rates when tested on culture positive sputa. For example, in the DS cohort the best performing mRNA target had a positivity rate of only 74% in culture positive sputum after initiation of therapy. Nonetheless, three mRNA targets did show positivity in culture negative sputa, highlighting their potential utility. For instance, positivity by both 16S rRNA and mRNA may predict patients at greatest risk of poor treatment outcomes.

In addition to the binary outcomes of positive versus negative, the 16S rRNA assay described here is also quantitative. Notably, 16S rRNA qRTPCR Ct values increased as Mtb numbers decreased in patients receiving first-line or second-line treatment regimens, highlighting its potential utility for monitoring treatment response in EBA trials (Fig. 4). The quantitative nature of the assay is underscored by the high coefficient of correlation found (median R = 0.88) when analyzing all sputa from this study (i.e. sputa from the two cohorts both before and after initiation of therapy; Fig. S13). Changes in coefficient of correlation and slope were observed for post-initiation of therapy sputa in some instances, which may reflect lingering 16S rRNA from dead or dying Mtb cells, the presence of certain viable but nonculturable Mtb populations that went undetected by our limiting dilution assay, and/or rifampin’s inhibition of Mtb RNA transcription.

The presence of rifampin, an Mtb RNA polymerase inhibitor, in first-line drug regimens may warrant caution in too closely correlating Mtb RNA levels with viability. We noticed a pattern in which 16S rRNA and mRNA targets showed lower positivity rates for culture positive sputa from the DS than DR cohort after, but not before, initiation of therapy. This was not on account of greater killing by first-line drugs: the median Mtb/mL for post-initiation of therapy sputa from the DS cohort was actually > two-fold higher than the DR cohort after two weeks of therapy. Rather, in vitro experiments revealed that rifampin and its corresponding inhibition of Mtb transcription may account for these differences (Fig. 6). Consistent with this hypothesis, analysis of one of the 16S rRNA primer sets (F1 R1 0.1 µM) on an expanded set of DS and DR sputa shows that the median qRTPCR Ct value, when normalized to Mtb numbers, was four-fold higher for post-initiation of therapy sputa from the DS than DR cohort (p = 0.002). Put another way, rifampin containing first-line therapies appear to reduce the abundance of 16S rRNA per viable Mtb cell in comparison to non-rifampin containing second-line therapies. Therefore, RNA-based assays may need to be interpreted differently when used on patients receiving rifamycin versus non-rifamycin based combination therapy.

We recognize the limitations of the current study. The clinical cohort sizes were relatively small and did not have power to make associations with TB treatment outcomes. Our goal was to develop an optimized RNA purification method and a panel of Mtb RNA markers that could be evaluated in future clinical trials. We also recognize that other promising RNA targets have been studied in the past, which we did not examine^15,16,18,32–35^. We propose that the most promising Mtb RNA candidates be tested in the large-scale clinical studies needed to establish associations between Mtb RNA in sputum and treatment outcomes^15–18,32–36^. Several factors distinguish the current study from previous ones: First, the method to purify RNA from sputum is novel and may be of use to other investigators studying Mtb in human sputum. Second, to account for cryptic Mtb populations that typically go undetected by standard culture methods, we included a limiting dilution assay capable of detecting differentially detectable/culturable Mtb. Finally, the performance of the assay was tested on the sputa of patients receiving rifamycin and non-rifamycin based therapy, allowing us to determine the effect of rifampin on the detection and quantification of Mtb RNA markers. The optimized Mtb RNA purification method and RNA markers outlined in this report are candidates for future clinical studies to correlate Mtb RNA detection and quantity with TB treatment response.

## Supplementary Information

Supplementary Information 1.

Supplementary Information 2.

Supplementary Information 3.

## Data Availability

All data generated during this study are included in this published article and its Supplementary Files.
