# Supplementary material for: An optimized method for purifying, detecting and quantifying Mycobacterium tuberculosis RNA from sputum for monitoring treatment response in TB patients
Source: Sci Rep. 2022 Oct 17;12:17382. doi: 10.1038/s41598-022-19985-w (PMC9574834; doi:10.1038/s41598-022-19985-w)
Supplement: Supplementary file 1 — Supplementary Information 1. [file 41598_2022_19985_MOESM1_ESM.docx]

**S1. Optimization experiments for the first pre-lysis step show that 0.5% n-lauryl sarcosine outperforms other detergents for reducing levels of host derived nucleic acids without negatively affecting Mtb RNA recovery.** Average qPCR cycle threshold values are presented from at least two replicates.

**S1.1**. Detergent 0.5% n-lauryl sarcosine most efficiently removes host RNAs (as measured by human actin) without negatively affecting Mtb RNA targets (16S rRNA and sigA mRNA).

|  | **PBS** | | | | | **Commercial** | |
| --- | --- | --- | --- | --- | --- | --- | --- |
| **Detergent:** | **—^*^** | **0.2% TYP** | **0.5% Triton X100** | **0.5% Tween 20** | **0.5% n-lauryl sarcosine** | **Prime-store** | **Qiagen RLT** |
| **16S rRNA**  **Avg Ct ± SD** | 20.0  ± .1 | 20.4  ± .1 | 21.4  ± .1 | 19.9  ± .1 | 19.7  ± .1 | 20.2  ± .1 | 20.5  ± .1 |
| **sigA mRNA**  **Avg Ct ± SD** | 31.0  ± .2 | 30.5  ± .1 | 32.1  ± .1 | 30.5  ± .1 | 30.1  ± .1 | 30.9  ± .3 | 31.2  ± .3 |
| **IS6110 DNA**  **Avg Ct ± SD** | 31.3  ± .4 | 30.8  ± .1 | 31.9  ± .1 | 30.8  ± .1 | 30.5  ± .1 | 31.6  ± .2 | 31.4  ± .4 |
| **Human actin**  **Avg Ct ± SD** | 26.6  ± .1 | 26.5  ± .1 | 29.7  ± .1 | 31.8  ± .1 | 34.1  ± .5 | 31.4  ± .1 | 32.4  ± .5 |

^*^ Dash mark indicates ‘none’ in this and subsequent tables; TYP, Tyloxapol; 0.3 mL of sputum was used

**S1.2.** Comparison of the detergent n-lauryl sarcosine with sodium dodecyl sulfate (SDS) for removing host RNAs without affecting Mtb RNA targets.

|  | **PBS + [n-lauryl sarcosine]** | | **PBS + [SDS]** | |
| --- | --- | --- | --- | --- |
|  | **0.5%** | **0.25%** | **0.5%** | **0.25%** |
| **16S rRNA**  **Avg Ct ± SD** | 9.5  ± .3 | 9.4  ± .3 | 9.6  ± .6 | 9.8  ± .5 |
| **sigA mRNA**  **Avg Ct ± SD** | 24.4  ± .2 | 24.2  ± .3 | 24.6  ± .4 | 24.5  ± .3 |
| **IS6110 DNA**  **Avg Ct ± SD** | 33.1  ± .2 | 32.8  ± .2 | 32.7  ± .1 | 32.4  ± .1 |
| **Human actin**  **Avg Ct ± SD** | 28.5  ± .3 | 25.9  ± .1 | 24.9  ± .4 | 24.0  ± .4 |

1 mL of sputum was used

**S1.3.** 0.5% n-lauryl sarcosine is superior to commercially available Qiagen RLT or RLT-plus buffers for removing host RNAs without affecting Mtb RNA targets.

|  | **PBS + [n-lauryl sarcosine]** | | | **Qiagen** | |
| --- | --- | --- | --- | --- | --- |
|  | **0.25%** | **0.5%** | **1%** | **RLT** | **RLT-plus** |
| **16S rRNA**  **Avg Ct ± SD** | 18.4  ± .1 | 18.6  ± .1 | 22.0  ± .1 | 20.0  ± .2 | 18.8  ± .2 |
| **sigA mRNA**  **Avg Ct ± SD** | 29.5  ± .1 | 29.6  ± .1 | 32.1  ± .7 | 30.0  ± .1 | 29.9  ± .1 |
| **IS6110 DNA**  **Avg Ct ± SD** | 30.6  ± .1 | 30.8  ± 3 | 32.8  ± .3 | 30.8  ± .5 | 31.7  ± .1 |
| **Human actin**  **Avg Ct ± SD** | 35.5  ± .5 | Undetec-table | 36.0* | 30.9  ± .1 | 31.1  ± .1 |

* Only 1/2 wells positive

**S1.4.** Combining 0.5% n-lauryl sarcosine with Qiagen RLT appears to negatively impact recovery of Mtb RNA targets.

|  | **PBS + 0.5% n-lauryl sarcosine** | | **RLT + 0.5% n-lauryl sarcosine** | |
| --- | --- | --- | --- | --- |
|  | **0% BM** | **1% BM** | **0% BM** | **1% BM** |
| **16S rRNA**  **Avg Ct ± SD** | 8.8  ± .1 | 7.9  ± .1 | 10.6  ± .4 | 11.5  ± .4 |
| **sigA mRNA**  **Avg Ct ± SD** | 24.1  ± .1 | 23.9  ± .1 | 26.1  ± .5 | 26.4  ± .6 |
| **85b mRNA**  **Avg Ct ± SD** | 23.2  ± .1 | 23.2  ± .1 | 25.5  ± .6 | 25.7  ± .9 |
| **Human actin**  **Avg Ct ± SD** | 23.3  ± .2 | 24.1  ± .1 | 27.0  ± 3 | 21.3  ± .4 |

BM, 2-mercaptoethanol

**S1.5.** Addition of up to 1% 2-mercaptoethanol (BM) further reduces recovery of human actin without affecting Mtb RNA targets.

|  | | **PBS 0.5% n-lauryl sarcosine** | | | | | | |  |
| --- | --- | --- | --- | --- | --- | --- | --- | --- | --- |
|  | | **0% BM** | | **0.1% BM** | | **0.33% BM** | | **1% BM** |  |
| **16S rRNA**  **Avg Ct ± SD** | | 14.0  ± .1 | | 13.9  ± .3 | | 13.6  ± .1 | | 14.0  ± .1 |  |
| **sigA mRNA**  **Avg Ct ± SD** | | 28.2  ± .3 | | 28.1  ± .1 | | 28.4  ± .1 | | 28.6  ± .1 |  |
| **IS6110 DNA**  **Avg Ct ± SD** | | 29.3  ± .1 | | 29.7  ± .2 | | 29.5  ± .1 | | 30.0  ± .1 |  |
| **Human actin**  **Avg Ct ± SD** | | 28.7  ± .2 | | 30.0  ± .1 | | 30.9  ± .1 | | 31.9  ± .5 |  |
|  | **PBS 0.5% n-lauryl sarcosine** | | | | | | | | |
|  | **0% BM** | | **0.5% BM** | | **1% BM** | | **2% BM** | | |
| **16S rRNA**  **Avg Ct ± SD** | 14.9  ± .1 | | 14.9  ± .3 | | 14.9  ± .1 | | 14.6  ± .1 | | |
| **sigA mRNA**  **Avg Ct ± SD** | 28.3  ± .3 | | 28.6  ± .3 | | 28.4  ± .2 | | 28.3  ± .1 | | |
| **IS6110 DNA**  **Avg Ct ± SD** | 28.7  ± .2 | | 28.7  ± .4 | | 28.6  ± .1 | | 28.5  ± .1 | | |
| **Human actin**  **Avg Ct ± SD** | 32.4  ± .3 | | 34.2  ± .9 | | 34.0  ± .7 | | 32.3  ± 3.2 | | |

BM, 2-mercaptoethanol; Samples were not DNase digested

**S1.6.** 0.5% n-lauryl sarcosine in PBS or 150mM NaCl pH 7.5-8 perform equivalently.

|  |  | **150mM NaCl 0.5% Sarcosine** | | **20mM Tris pH 7.5**  **0.5% Sarcosine** | | |
| --- | --- | --- | --- | --- | --- | --- |
|  | **PBS 0.5% Sarcosine 1% BM** | **20mM Tris pH 8** | **20mM MES pH 6** | **75mM NaCl**  **1% BM** | **150mM NaCl**  **1% BM** | **300mM NaCl**  **1% BM** |
| **16S rRNA**  **Avg Ct ± SD** | 14.1  ± .3 | 14.0  ± .9 | 13.6  ± .3 | 13.1  ± .1 | 12.9  ± .2 | 13.2  ± .2 |
| **sigA mRNA**  **Avg Ct ± SD** | 26.3  ± .2 | 26.5  ± .2 | 27.1  ± .3 | 26.2  ± .2 | 26.1  ± .3 | 26.4  ± .3 |
| **IS6110 DNA**  **Avg Ct ± SD** | 36.5  ± 1.1 | Undetec-table | 37.2* | 36.0  ± 1.1 | 35.9  ± .3 | Undetec-table |
| **Human actin**  **Avg Ct ± SD** | 34.2  ± .2 | 34.1  ± .5 | 33.5  ± .6 | 33.4  ± .3 | 34.1  ± .4 | 33.8* |

* Only 1/2 wells positive; Sarcosine, n-lauryl sarcosine; BM, 2-mercaptoethanol

**S1.7.** 0.5% n-lauryl sarcosine outperforms commercially available RIPA lysis buffer.

|  |  | **RIPA buffer^1^** | | | |
| --- | --- | --- | --- | --- | --- |
|  | **0.5% Sarcosine PBS** | **2X** | **1X** | **2X** | **1X** |
| **BM:** | **1%** | **0%** | **0%** | **1%** | **1%** |
| **16S rRNA**  **Avg Ct ± SD** | 15.7 ± .1 | 15.4 ± .2 | 15.8 ± .1 | 15.2 ± .1 | 15.5 ± .1 |
| **sigA mRNA**  **Avg Ct ± SD** | 29.1 ± .1 | 28.8 ± .1 | 29.2 ± .1 | 28.6 ± .1 | 29.0 ± .1 |
| **IS6110 DNA**  **Avg Ct ± SD** | 33.7 ± .2 | 35.8* | 35.3* | 37.4* | 34.4 ± .1 |
| **Human actin**  **Avg Ct ± SD** | 32.2 ± .3 | 28.3 ± .1 | 26.2 ± .1 | 28.7 ± .1 | 25.7 ± .1 |

^1^ 1X RIPA: 50mM Tris HCl ph 7.4, 150mM NaCl, 1% NP-40, 0.5% sodium deoxycholate, 0.1% SDS and 1mM EDTA; BM, 2-mercaptoethanol; Sarcosine, n-lauryl sarcosine; * Only 1/2 wells positive

**S2. Adding a second prelysis wash (pre-lysis 2) removes more host derived nucleic acids and Mtb DNA than a single prelysis step without negatively impacting (and at times improving) recovery of Mtb RNAs.** Average qPCR cycle threshold values are presented from at least two replicates.

**S2.1**. Inclusion of two prelysis washes removes a substantial portion of host RNAs and Mtb DNA while also improving recovery of Mtb RNA.

| **Pre-lysis 1:** | **PBS 0.5% Sarcosine 1% BM** | |
| --- | --- | --- |
| **Pre-lysis 2:** | **PBS** | **RLT** |
| **16S rRNA**  **Avg Ct ± SD** | 10.8  ± .1 | 9.5  ± .5 |
| **sigA mRNA**  **Avg Ct ± SD** | 25.1  ± .2 | 24.4  ± .2 |
| **IS6110 DNA**  **Avg Ct ± SD** | 28.9  ± .1 | 33.1  ± .2 |
| **Human actin**  **Avg Ct ± SD** | 23.8  ± .1 | 33.1  ± .2 |

Sarcosine, n-lauryl sarcosine; BM, 2-mercaptoethanol

**S2.2.** Inclusion of Qiagen RLT as a second pre-lysis buffer removes more host derived RNAs (as measured by qRTPCR for human actin) and Mtb DNA (as measured by IS6110) while also improving recovery of Mtb mRNA targets (sigA mRNA).

| **Pre-lysis 1:** | **PBS 0.5% Sarcosine 1% BM** | | | |
| --- | --- | --- | --- | --- |
| **Pre-lysis 2:** | **5mM Tris ph 7** | **500mM NaCl** | **1M NaCl** | **Qiagen RLT** |
| **16S rRNA**  **Avg Ct ± SD** | 11.9  ± .4 | 10.7  ± .8 | 10.8  ± .9 | 10.1  ± 1.0 |
| **sigA mRNA**  **Avg Ct ± SD** | 25.8  ± .1 | 25.1  ± .2 | 25.4  ± .3 | 24.8  ± .1 |
| **IS6110 DNA**  **Avg Ct ± SD** | 26.8  ± .1 | 31.3  ± .2 | 31.2  ± .1 | 30.3  ± .1 |
| **Human actin**  **Avg Ct ± SD** | 23.4  ± .2 | 25.2  ± .6 | 23.1  ± .7 | 29.4  ± .6 |

Sarcosine, n-lauryl sarcosine; BM, 2-mercaptoethanol

**S2.3.** Inclusion of Qiagen RLT as a second prelysis wash removes more host RNAs while improving recovery of Mtb RNA.

| **Pre-lysis 1:** | **PBS 0.5% Sarcosine 1% BM** | | **PBS 0.25% Sarcosine 1% BM** | |
| --- | --- | --- | --- | --- |
| **Pre-lysis 2:** | **PBS** | **RLT** | **PBS** | **RLT** |
| **16S rRNA**  **Avg Ct ± SD** | 10.8  ± .1 | 9.5  ± .5 | 17.9  ± .3 | 9.4  ± .3 |
| **sigA mRNA**  **Avg Ct ± SD** | 25.1  ± .2 | 24.4  ± .2 | 29.4  ± .1 | 24.2  ± .3 |
| **IS6110 DNA**  **Avg Ct ± SD** | 28.9  ± .1 | 33.1  ± .2 | 29.1  ± .1 | 32.8  ± .2 |
| **Human actin**  **Avg Ct ± SD** | 23.8  ± .1 | 33.1  ± .2 | 25.2  ± .2 | 25.9  ± .1 |

Sarcosine, n-lauryl sarcosine; BM, 2-mercaptoethanol

**S2.4.** Inclusion of 2-mercaptoethanol (BM) in prelysis-2 (RLT buffer) does not further improve removal of host nucleic acids.

| **Pre-lysis 1:** | **PBS 0.5% n-lauryl sarcosine 1% BM** | | |
| --- | --- | --- | --- |
| **Pre-lysis 2:** | **RLT** | | |
|  | **0% BM** | **0.5% BM** | **1% BM** |
| **16S rRNA**  **Avg Ct ± SD** | 10.4  ± .3 | 10.0  ± .2 | 10.9  ± .2 |
| **sigA mRNA**  **Avg Ct ± SD** | 23.0  ± .1 | 22.7  ± .1 | 23.6  ± .1 |
| **IS6110 DNA**  **Avg Ct ± SD** | 34.8  ± .4 | 32.3  ± .1 | 32.3  ± .1 |
| **Human actin**  **Avg Ct ± SD** | 31.6  ± .7 | 30.5  ± .3 | 30.9  ± .1 |

BM, 2-mercaptoethanol

**S2.5.** Use of a three step prelysis procedure removes the most host-derived RNAs and may be particularly useful for assays such as RNAseq which are sensitive to contamination by host nucleic acids.

| **Pre-lysis 1:** | **PBS 0.5% Sarcosine 1% BM** | | | | | |
| --- | --- | --- | --- | --- | --- | --- |
| **Pre-lysis 2:** | **RLT** | **PBS 0.5% Sarcosine** | | **PBS 0.25% Sarcosine** | | **RLT-plus** |
| **Pre-lysis 3:** | **—** | **PBS** | **RLT** | **PBS** | **RLT** | **RLT** |
| **16S rRNA**  **Avg Ct ± SD** | 10.5 ± .2 | 10.6 ± .2 | 12.1 ± .1 | 10.3 ± .3 | 11.8 ± .1 | 11.2 ± .2 |
| **sigA mRNA**  **Avg Ct ± SD** | 24.6 ± .1 | 24.2 ± .1 | 25.7 ± .1 | 23.9 ± .1 | 25.4 ± .1 | 24.9 ± .1 |
| **IS6110 DNA**  **Avg Ct ± SD** | 30.4 ± .1 | 31.6 ± .2 | 31.6 ± .2 | 29.8 ± .3 | 31.3 ± .7 | 30.7 ± .1 |
| **Human actin**  **Avg Ct ± SD** | 28.5 ± .2 | 29.1 ± .2 | 32.2 ± .2 | 28.0 ± .3 | 31.9 ± .1 | 33.9 ± .2 |

Sarcosine, n-lauryl sarcosine; BM, 2-mercaptoethanol; Samples were not DNase digested

| **Pre-lysis 1:** | **PBS 0.5% n-lauryl sarcosine 1% BM** | | | |
| --- | --- | --- | --- | --- |
| **Pre-lysis 2:** | **RLT** | **PBS 0.5% Sarcosine** | **PBS 0.5% Sarcosine 0.5% BM** | **RLT-plus** |
| **Pre-lysis 3:** | **—** | **PBS** | **PBS** | **PBS** |
| **16S rRNA**  **Avg Ct ± SD** | 10.4 ± .3 | 10.6 ± .2 | 9.5 ± .3 | 10.7 ± .1 |
| **sigA mRNA**  **Avg Ct ± SD** | 25.0 ± .1 | 25.1 ± .1 | 24.3 ± .1 | 25.9 ± .2 |
| **IS6110 DNA**  **Avg Ct ± SD** | 32.9 ± .4 | 33.4 ± .1 | 32.1 ± .3 | 34.8 ± .1 |
| **Human actin**  **Avg Ct ± SD** | 27.7 ± .1 | 29.1 ± .1 | 28.9 ± .1 | 34.5 ± .1 |

Sarcosine, n-lauryl sarcosine; BM, 2-mercaptoethanol

**S3. Guanidine thiocyanate containing lysis buffers work efficiently for bead beating.** Average qPCR cycle threshold values are presented from at least two replicates.

**S3.1.** Qiagen RLT and 4M guanidine thiocyanate (GuSCN) perform equivalently.

| **Lysis 1:** | **RLT** | **4M GuSCN pH 6** | **4M GuSCN pH 7** | **4M GuSCN pH 8** |
| --- | --- | --- | --- | --- |
| **16S rRNA**  **Avg Ct ± SD** | 15.5  ± 1.4 | 16.8  ± .1 | 14.9  ± .3 | 15.4  ± .3 |
| **sigA mRNA**  **Avg Ct ± SD** | 29.2  ± .2 | 31.1  ± .3 | 28.8  ± .1 | 28.6  ± .1 |
| **IS6110 DNA**  **Avg Ct ± SD** | 29.5  ± .1 | 30.6  ± .1 | 28.9  ± .1 | 26.9  ± .1 |
| **Human actin**  **Avg Ct ± SD** | 25.7  ± .1 | 28.6  ± .1 | 25.6  ± .3 | 25.9  ± .2 |

Samples were not DNase digested

**S3.2.** Addition of 2-mercaptoethanol (BM) does not further improve the performance of RLT for bead beating.

| **Lysis 1:** | **RLT** | | |
| --- | --- | --- | --- |
| **BM:** | **0%** | **0.1%** | **0.5%** |
| **16S rRNA**  **Avg Ct ± SD** | 10.5  ± .3 | 11.1  ± .4 | 10.9  ± .4 |
| **sigA mRNA**  **Avg Ct ± SD** | 22.9  ± .1 | 22.8  ± .5 | 23.0  ± .4 |
| **IS6110 DNA**  **Avg Ct ± SD** | 33.6  ± .4 | 33.5  ± .1 | 33.8  ± .3 |
| **Human actin**  **Avg Ct ± SD** | 30.7  ± .2 | 30.8  ± .5 | 30.6  ± .3 |

**S4. Control experiments with different protocols for lysis of Mtb cells.** Average qPCR cycle threshold values are presented from at least two replicates.

**S4.1** Bead beating outperforms other lysis methods such as heat, proteinase K and lysozyme M treatment for recovery of Mtb RNA**.** Bead beating with commercially available bead beating tubes outperform using 500µL .1mm zirconium beads.

|  | **- Bead beating** | | | | | **+ Bead beating** | | | |
| --- | --- | --- | --- | --- | --- | --- | --- | --- | --- |
|  | **RT** | **65C for 2 hours in RLT-plus lysis buffer:** | | | | **Beading beating with:** | | | |
|  | **—** | **—** | **Prot K** | **Lys M** | **Prot K + Lys M** | **500µL 0.1mm Zirconium** | **Matrix B** | **Beadbug 0.1mm**  **Silica** | **Beadbug 0.1mm Zirconium** |
| **16S rRNA**  **Avg Ct ± SD** | 27.8  ± .4 | 29.8  ± .1 | 27.5  ± .1 | 28.2  ± .2 | 27.5  ± .1 | 20.0  ± .1 | 19.2  ± .2 | 19.3  ± .3 | 18.7  ± .1 |
| **icl1 mRNA**  **Avg Ct ± SD** | 31.3  ± .3 | 34.1  ± .4 | 31.0  ± .2 | 32.0  ± .1 | 30.9  ± .1 | 26.0  ± .2 | 23.9  ± .2 | 23.6  ± .1 | 23.1  ± .1 |

RT, room temperature; Prot K, proteinase K; Lys M, lysozyme M; Samples were not DNase digested

**S4.2** Commercially available pre-filled bead beating tubes appear to work better than using 500µL 0.1mm zirconium beads.

|  | **Beading beating with:** | | | |
| --- | --- | --- | --- | --- |
|  | **500µL 0.1mm Zirconium** | **Matrix B** | **Beadbug 0.1mm**  **Silica** | **Beadbug 0.1mm Zirconium** |
| **icl1 mRNA**  **Avg Ct ± SD** | 27.0  ± .4 | 24.6  ± .3 | 24.3  ± .1 | 24.3  ± .4 |
| **sigA mRNA**  **Avg Ct ± SD** | 24.1  ± .2 | 21.7  ± .3 | 21.2  ± .4 | 21.2  ± .2 |

Samples were not DNase digested

**S4.3** Bead beating three times (30 seconds each) with chilling in between is sufficient to lyse Mtb cells.

|  | **Bead beating** | | | |
| --- | --- | --- | --- | --- |
|  | **30 sec x 3** | **45 sec x 3** | **60 sec x 3** | **60 sec x 4** |
| **16S rRNA**  **Avg Ct ± SD** | 21.0  ± .3 | 21.8  ± .2 | 22.2  ± 1.1 | 22.0  ± .4 |
| **IS6110 DNA**  **Avg Ct ± SD** | 18.8  ± .2 | 19.4  ± .1 | 19.0  ± .8 | 19.3  ± .6 |

Samples were not DNase digested

|  | **Bead beating** | | |
| --- | --- | --- | --- |
|  | **30 sec x 3** | **60 sec x 2** | **60 sec x 3** |
| **16S rRNA**  **Avg Ct ± SD** | 26.9  ± .2 | 27.2  ± .3 | 26.8  ± .2 |
| **IS6110 DNA**  **Avg Ct ± SD** | 29.3  ± .1 | 29.8  ± .2 | 28.6  ± .1 |

Samples were not DNase digested

**S5. Testing of different lysis-2 buffers shows that adding RLT-plus (or RLT buffer with detergent) after bead beating helps to recover more Mtb RNA.** Average qPCR cycle threshold values are presented from at least two replicates.

**S5.1** Using a lower volume of lysis-1 buffer Qiagen RLT (500µL) for bead beating is superior to using a higher volume (900µL).

| **Lysis 1:** | **900µL RLT** | **500µL RLT** | |
| --- | --- | --- | --- |
| **Lysis 2:** | **—** | **400µL RLT** | **400µL RLT-plus** |
| **16S rRNA**  **Avg Ct ± SD** | 11.9  ± .1 | 10.0  ± .1 | 9.7  ± .1 |
| **sigA mRNA**  **Avg Ct ± SD** | 26.2  ± .1 | 24.8  ± .1 | 24.3  ± .1 |
| **IS6110 DNA**  **Avg Ct ± SD** | 32.0  ± .1 | 27.1  ± .1 | 28.2  ± .1 |
| **Human actin**  **Avg Ct ± SD** | 26.0  ± .2 | 24.8  ± .1 | 24.3  ± .1 |

Samples were not DNase digested

**S5.2** RLT-plus performs equivalently to RLT with addition of different detergents for lysis 2.

| **Lysis 1:** | **500µL RLT** | | | |
| --- | --- | --- | --- | --- |
| **Lysis 2:** | **400µL RLT-plus** | **400µL RLT 1% Triton** | **400µL RLT 1% Tween 20** | **400µL RLT 0.5% Sarcosine** |
| **16S rRNA**  **Avg Ct ± SD** | 10.5  ± .3 | 10.8  ± .1 | 11.0  ± .3 | 10.9  ± .2 |
| **sigA mRNA**  **Avg Ct ± SD** | 22.9  ± .1 | 23.0  ± .4 | 23.4  ± .1 | 23.1  ± .4 |
| **IS6110 DNA**  **Avg Ct ± SD** | 33.6  ± .4 | 33.1  ± .1 | 33.4  ± .3 | 33.3  ± .2 |
| **Human actin**  **Avg Ct ± SD** | 30.7  ± .2 | 31.3  ± .1 | 31.4  ± .2 | 31.1  ± .5 |

Sarcosine, n-lauryl sarcosine

**S6. Testing of various different DNA/RNA spin columns shows that Omega Biotek Hibind DNA spin columns work equivalently to most other commercial DNA/RNA spin columns for removal of DNA without affecting Mtb RNA.** Average qPCR cycle threshold values are presented from at least two replicates.

**S6.1.** Combination of different DNA and RNA spin columns for removal of DNA (as measured by IS6110) and purification of RNA show equivalent performance.

| **DNA column:** | **DNA Hibind** | **DNA Hibind (2x cap.)** | **RNA Hibind** | **DNA Hibind** | **DNA Hibind** | **Zymo IIIcg** |
| --- | --- | --- | --- | --- | --- | --- |
| **RNA column:** | **RNA Hibind** | **RNA Hibind** | **RNA Hibind** | **DNA Hibind** | **Zymo IIIcg** | **Zymo IIIcg** |
| **16S rRNA**  **Avg Ct ± SD** | 17.7  ± .2 | 16.4  ± .1 | 16.6  ± .1 | 15.9  ± .1 | 17.4  ± .1 | 17.4  ± .1 |
| **sigA mRNA**  **Avg Ct ± SD** | 27.8  ± .1 | 27.6  ± .1 | 27.6  ± .1 | 27.4  ± .1 | 28.1  ± .1 | 28.6  ± .1 |
| **icl1 mRNA**  **Avg Ct ± SD** | 28.1  ± .2 | 27.8  ± .1 | 28.0  ± .1 | 27.8  ± .1 | 28.6  ± .1 | 29.0  ± .1 |
| **IS6110 DNA**  **Avg Ct ± SD** | 29.0  ± .2 | 28.5  ± .1 | 28.7  ± .1 | 28.6  ± .1 | 29.3  ± .1 | 29.4  ± .1 |

Samples were not DNase digested

| **Sputum volume extracted:** | **333µL** | | **1mL** | |
| --- | --- | --- | --- | --- |
| **DNA column:** | **Hibind DNA** | **Hibind DNA (2x cap.)** | **Hibind DNA** | **Hibind DNA (2x cap.)** |
| **16S rRNA**  **Avg Ct ± SD** | 14.6  ± .1 | 14.6  ± .1 | 12.8  ± .1 | 13.0  ± .2 |
| **sigA mRNA**  **Avg Ct ± SD** | 28.5  ± .1 | 28.9  ± .2 | 27.3  ± .3 | 27.2  ± .2 |
| **IS6110 DNA**  **Avg Ct ± SD** | 29.3  ± .1 | 29.3  ± .1 | 27.6  ± .2 | 27.8  ± .1 |
| **Human actin**  **Avg Ct ± SD** | 26.0  ± .2 | 26.3  ± .3 | 24.1  ± .1 | 25.0  ± .1 |

**S6.2.** Use of a Hibind DNA column with 2-fold higher DNA binding capacity does not improve removal of Mtb DNA from sputum for volumes up to 1mL.

Samples were not DNase digested

**S7. Addition of alcohol (final concentration v/v) to the DNA spin column flow through improves binding of Mtb RNA to the RNA spin column.** Average qPCR cycle threshold values are presented from two replicates.

**S7.1** Addition of ≥145µL of isopropanol (ISOH) or ethanol (ETOH) to the DNA spin column flow through shows the best recovery of Mtb RNA target 16S rRNA.

| **Alcohol:** | **100µL ISOH (12.5%)** | **100µL ETOH**  **(12.5%)** | **145µL ISOH (17%)** | **145µL ETOH (17%)** | **250µL ISOH (25%)** | **250µL ETOH (25%)** |
| --- | --- | --- | --- | --- | --- | --- |
| **16S rRNA**  **Avg Ct ± SD** | 16.2  ± .1 | 16.7  ± .2 | 15.3  ± .1 | 15.7  ± .1 | 15.7  ± .1 | 15.6  ± .3 |
| **IS6110 DNA**  **Avg Ct ± SD** | 26.6  ± .1 | 27.6  ± .2 | 27.8  ± .1 | 28.1  ± .1 | 28.1  ± .1 | 27.6  ± .1 |

Samples were not DNase digested

**S8. Omega Biotek Hibind RNA spin columns work equivalently to most other commercially available spin columns for purification of Mtb RNA (with the exception of Qiagen DNeasy).** If a more concentrated eluate is required, Hibind Microelute spin columns allows for elution in a lower volume (20µL versus 50µL). Average qPCR cycle threshold values are presented from at least two replicates.

| **Company:** | **Omega Biotek** | | **Zymo Research** | | **Qiagen** | |
| --- | --- | --- | --- | --- | --- | --- |
| **RNA column:** | **Hibind RNA** | **Hibind DNA** | **IIcr** | **IIIcg** | **RNeasy** | **DNeasy** |
| **16S rRNA**  **Avg Ct ± SD** | 18.2  ± .1 | 18.2  ± .1 | 18.7  ± .1 | 18.8  ± .1 | 18.4  ± .1 | 21.2  ± .1 |
| **icl1 mRNA**  **Avg Ct ± SD** | 19.3  ± .1 | 19.3  ± .1 | 20.4  ± .1 | 20.8  ± .1 | 20.5  ± .1 | 27.9  ± .1 |

|  | **Omega Biotek** | **Qiagen** | **Biovision** | **Norgen** | **Zymo Research** | |
| --- | --- | --- | --- | --- | --- | --- |
| **RNA column:** | **Hibind RNA** | **RNeasy** | **Sputum RNA** | **Sputum RNA** | **Zymo III** | **Zymo IIIcg** |
| **16S rRNA**  **Avg Ct ± SD** | 19.2  ± .3 | 20.4  ± .1 | 19.5  ± .1 | 19.2  ± .1 | 20.3  ± .1 | 19.4  ± .2 |
| **icl1 mRNA**  **Avg Ct ± SD** | 27.8  ± .1 | 27.2  ± .1 | 27.8  ± .1 | 29.3  ± .1 | 28.8  ± .1 | 27.1  ± .1 |

| **RNA column:** | **Hibind RNA** | **Hibind Microelute** |
| --- | --- | --- |
| **Elution volume:** | **50µL** | **20µL** |
| **16S rRNA**  **Avg Ct ± SD** | 17.7  ± .2 | 15.1  ± .3 |
| **sigA mRNA**  **Avg Ct ± SD** | 27.8  ± .1 | 26.5  ± .1 |
| **icl1 mRNA**  **Avg Ct ± SD** | 28.1  ± .2 | 26.6  ± .1 |
| **IS6110 DNA**  **Avg Ct ± SD** | 29.0  ± .2 | 27.5  ± .2 |

Samples were not DNase digested

**S9. Wash 1 buffers RLT/RLT-plus with 17% isopropanol work equivalently.** Average qPCR cycle threshold values are presented from two replicates.

| **Wash 1**: | **17% ISOH** | | |
| --- | --- | --- | --- |
|  | **RLT** | **RLT:RLT-plus (1:1)** | **RLT-plus** |
| **16S rRNA**  **Avg Ct ± SD** | 13.4  ± .3 | 13.1  ± .1 | 13.4  ± .1 |
| **sigA mRNA**  **Avg Ct ± SD** | 27.5  ± .2 | 27.3  ± .1 | 27.4  ± .1 |
| **IS6110 DNA**  **Avg Ct ± SD** | 35.5  ± .3 | 37.1  ± .7 | 35.6* |
| **Human actin**  **Avg Ct ± SD** | 31.2  ± .1 | 31.2  ± .1 | 31.1  ± .4 |

* Only 1/2 wells positive

**S10.** **Wash 2 buffers consisting of 70%-80% ethanol pH 7-8 work best for recovery of Mtb RNA as indicated by lower cycle threshold values for Mtb 16S rRNA and sigA mRNA.** Average qPCR cycle threshold values are presented from three replicates.

| **Buffer:** | **10mM Tris pH 8** | | **10mM Tris pH 7** | | **20% PBS** | **30% PBS** | **50% PBS** | **100mM NaCl pH 7.5** | — |
| --- | --- | --- | --- | --- | --- | --- | --- | --- | --- |
| **Alcohol:** | **80% ETOH** | **70% EOTH** | **80% ETOH** | **70% ETOH** | **80% ETOH** | **70% ETOH** | **25% ETOH 25% ISOH** | **25% ETOH 25% ISOH** | **80% ETOH** |
| **16S rRNA**  **Avg Ct ± SD** | 13.3  ± .1 | 13.1  ± .1 | 13.3  ± .1 | 13.7  ± .1 | 14.0  ± .2 | 14.1  ± .1 | 15.0  ± .5 | 15.1  ± .3 | 13.2  ± .2 |
| **sigA mRNA**  **Avg Ct ± SD** | 27.5  ± .1 | 27.5  ± .1 | 27.1  ± .1 | 27.2  ± .1 | 27.7  ± .3 | 28.2  ± .4 | 29.5  ± .1 | 29.2  ± .2 | 27.3  ± .1 |
| **IS6110 DNA**  **Avg Ct ± SD** | 36.8  ± 1.4 | 36.2  ± 1.1 | 36.6  ± 1.7 | 36.9  ± .6 | 35.2  ± .5 | 37.4  ± .3 | 35.6  ± 1.0 | 36.5  ± .3 | 35.3  ± .1 |
| **Human actin**  **Avg Ct ± SD** | 31.5  ± .8 | 31.2  ± .1 | 31.3  ± .4 | 31.6  ± .2 | 30.9  ± .4 | 31.7  ± .4 | 31.9  ± .2 | 32.8  ± .4 | 31.1  ± .1 |

ETOH, ethanol; ISOH, isopropanol

**S11. Control experiments showing effects of DNase treatment at room temperature (RT) or 37C on Mtb DNA (IS6110) and RNA targets (16S rRNA and sigA mRNA).** Average qPCR cycle threshold values are presented from two replicates.

|  | **- DNase** | **+ DNase** | | | | |
| --- | --- | --- | --- | --- | --- | --- |
|  | **RT**  **40min** | **RT**  **20min** | **RT**  **30min** | **RT**  **40min** | **37C* 10min** | **37C* 20min** |
| **16S rRNA**  **Avg Ct ± SD** | 13.5  ± .1 | 13.3  ± .1 | 13.3  ± .1 | 13.4  ± .1 | 13.5  ± .1 | 13.5  ± .4 |
| **sigA mRNA**  **Avg Ct ± SD** | 26.6  ± .1 | 26.7  ± .1 | 26.7  ± .1 | 26.7  ± .1 | 26.8  ± .1 | 26.6  ± .1 |
| **IS6110 DNA**  **Avg Ct ± SD** | 26.3  ± .1 | 31.4  ± .2 | 32.4  ± .4 | 34.3  ± .3 | 30.4  ± .1 | 33.0  ± .2 |
| **Human actin**  **Avg Ct ± SD** | 27.1  ± .1 | 30.6  ± .1 | 31.2  ± .2 | 31.0  ± .1 | 30.4  ± .2 | 31.0  ± .2 |

* Tubes kept in 37C incubator (not heat plate)


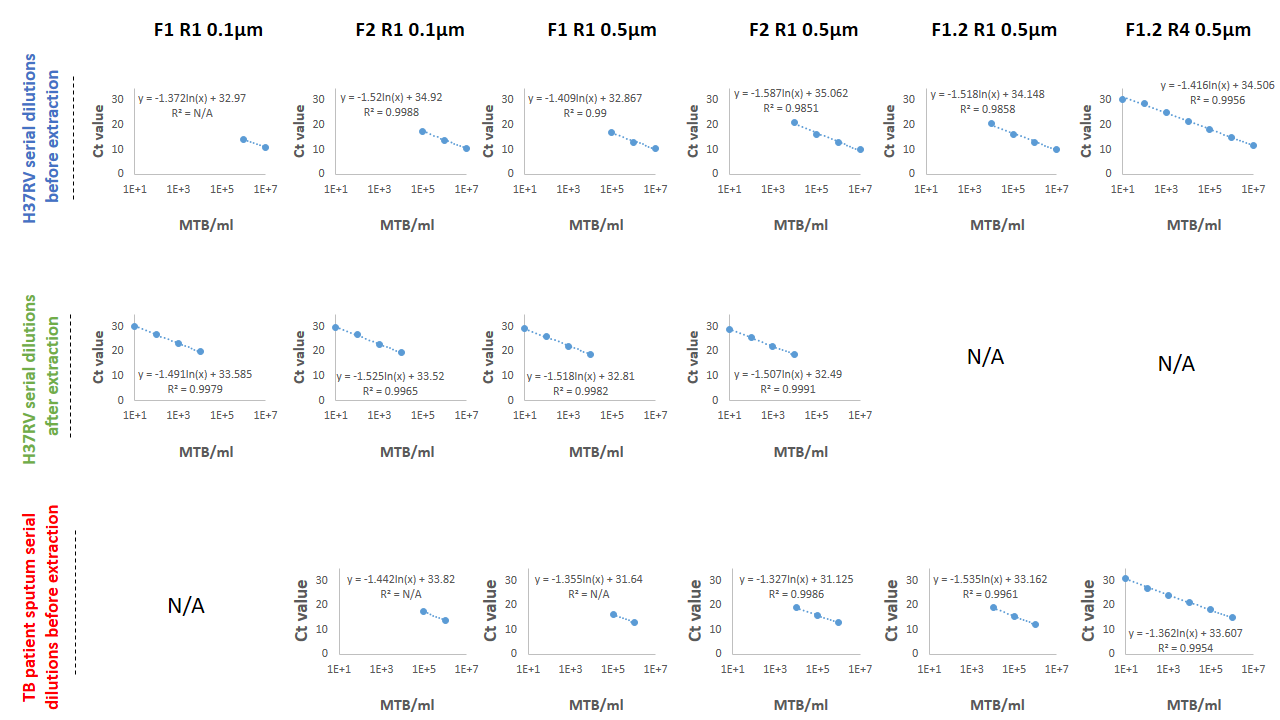


**S12.** Standard curves for each 16S rRNA primer set obtained with serial dilutions of H37Rv (10^1^-10^7^ Mtb/mL) spiked into non-MTB containing sputum. Top row indicates serial dilutions that were made prior to extraction (meaning H37Rv Mtb was diluted and then RNA extracted), middle row indicates serial dilutions of H37Rv RNA made after extraction (meaning H37Rv RNA was extracted and then the RNA was serially diluted), and bottom row represents serial dilution of a TB patient sputum sample made before extraction using non-Mtb containing sputum (10^1^-10^6^ Mtb/mL). The final primer set (F1.2 R4 0.5µM) has the lowest LoD of any primer set tested but is unable to distinguish between live/dead H37Rv (Table 4).


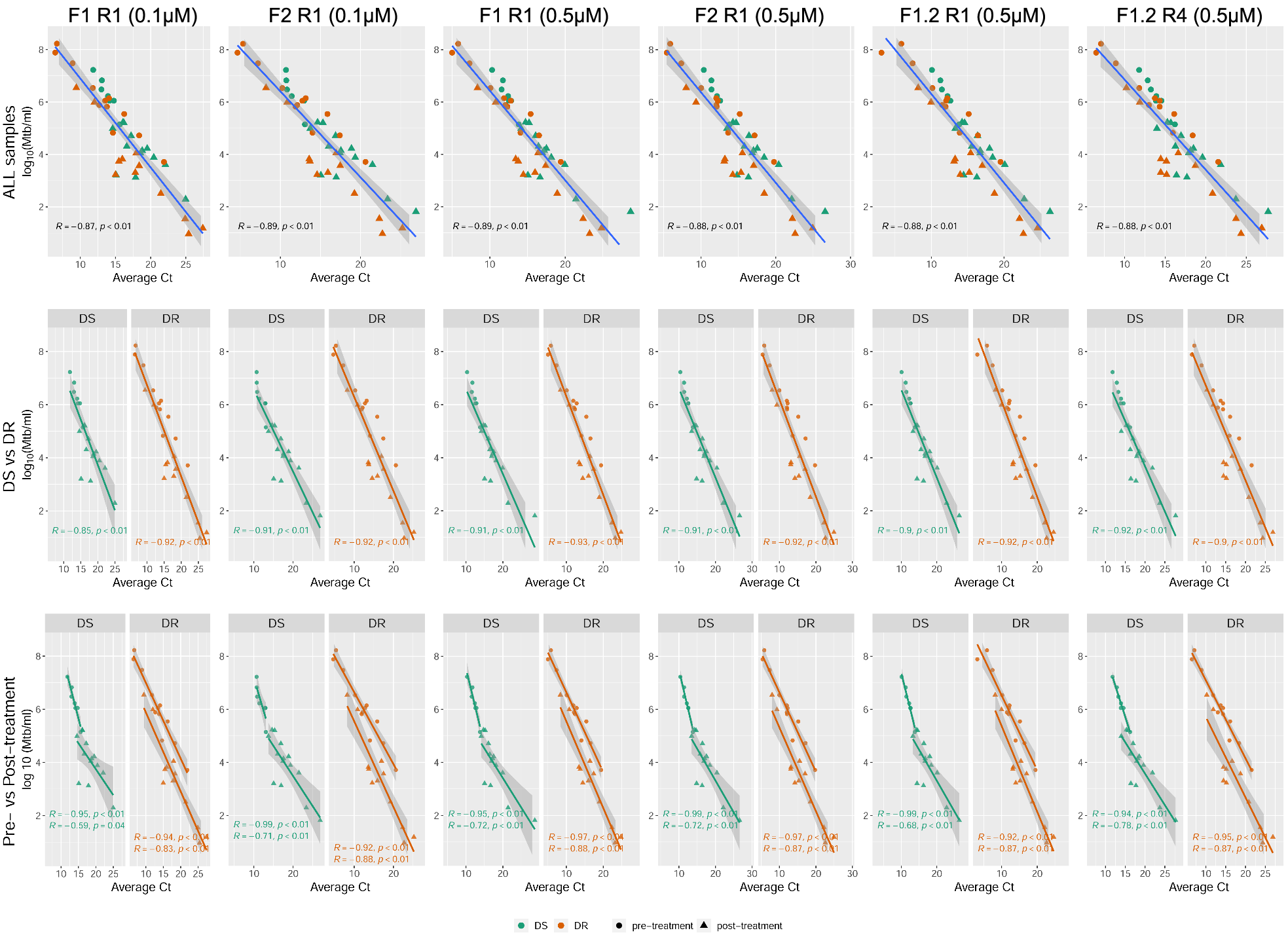


**S13. Standard curves for the 16S rRNA primer sets for: all sputa (from both cohorts at both timepoints) (top row); subjects with drug sensitive (DS) or drug resistant (DR) TB (middle row); or pre- versus post-initiation of treatment samples (bottom row). The final primer set (F1.2 R4 0.5µM) has the lowest LoD of any primer set tested but is unable to distinguish between live/dead H37Rv (Table 4).**

**S14 Appendix. Protocol for RNA extraction for qRTPCR from neat sputum.**

|  | **Commercial**  **Buffers** | **Homemade**  **Buffer Substitutes** |
| --- | --- | --- |
| **Pre-lysis 1:** | PBS  0.5% n-lauryl sarcosine  1% 2-mercaptoethanol | 150mM NaCl  20mM Tris pH 7.4  0.5% n-lauryl sarcosine  1% 2-mercaptoethanol |
| **Pre-lysis 2*:** | RLT | 4M Guanidine thiocyanate |
| **Lysis 1*:** | RLT | 4M Guanidine thiocyanate |
| **Lysis 2*:** | RLT-plus | 4M Guanidine thiocyanate  1% Triton |
| **Wash 1*:** | RLT  16.7% ISOH | 4M Guanidine thiocyanate  16.7% ISOH |
| **Wash 2^1^:** | 80% ETOH  10mM Tris pH 7 | 80% ETOH  10mM Tris pH 7 |
| **Elution:** | 10mM Tris pH 8 | 10mM Tris pH 8 |

**NOTE:**

- This protocol needs to performed in a **biosafety level 3** laboratory with appropriate biosafety protocols and personal protective equipment including **N95 mask**

- A list of all reagents and sources can be found at **S17 Appendix**

***** These buffers contain guanidine thiocyanate which is INCOMPATIBLE with bleach

**Protocol**

1. Transfer up to 25mL of neat sputum into 50mL tube, screw lid tightly
   1. Rigorously vortex at maximum setting for up to 5min or until homogenized^2^
2. Let tube sit for 5min (to allow aerosols to settle), then pipette 333µL into 3 screwcap 1.7mL tubes^3^
   1. Use wide orifice 1mL tips
3. Pipette 666µL of **pre-lysis 1** into each of the three tubes
   1. Pipette up/down 10 times (use non-wide orifice 1mL tip), close lid tightly, vortex for 30 sec
4. Spin at max speed for 5min
5. Remove supernatant, resuspend the 3 pellets with 1mL of **pre-lysis 2** and combine into 1 tube
6. Spin at maximum speed for 5min, remove supernatant
7. Resuspend pellet with 500µL of **lysis 1** and transfer to a pre-chilled Beadbeater Zirconium tube
8. Bead beat for 30sec, chill for 1 min, repeat two more times
9. Spin max speed for 15sec
10. Add 400µL **lysis 2** into the tube, vortex, spin max speed 15sec
11. Transfer 750µL of supernatant into Omega DNA^4^ spin column
12. Spin max speed 30sec^5^
13. Add 145µL isopropanol to flow-through, mix by pipetting up/down and transfer 750µL to Omega RNA spin column^4^
14. Spin max speed 30 sec
15. Transfer column to new 2mL capless tube, pipette 500µL **Wash 1** to RNA column
16. Spin max speed 30sec
17. Transfer column to new 2mL capless tube, pipette 500µL **Wash 2** to RNA column
18. Spin max speed 1min
19. Transfer column to new 1.7mL tube, place in 60-65C warm plate for 5min to dry
20. Elute with 50µL **elution** buffer (pre-warm to 60-65C if you have many tubes)
21. Spin max speed for 1min
22. Add 5µL of 10X DNase buffer and 4µL of DNase (mix by tapping, DO NOT VORTEX), spin down
23. Incubate at 37C for 30min
24. Add 2µL 50mM EDTA, vortex vigorously
25. Incubate 65C for 10min
26. Spin down max speed, freeze at -80C

^1^ If eluting with H_2_0 (which is not recommended due to lower yield), use 80% ETOH 10mM Tris pH 8

^2^ Expectorated samples require more effort to homogenize, invert before vortexing

^3^ Samples can be frozen at -80C at this step, freeze in 1.2mL aliquots in screw cap tubes

^4^  Other DNA/RNA columns are compatible though the protocol has been optimized with Omega Biotek

^5^ DNA column can be saved if DNA is required (see **S16 Appendix**) or discarded if not

**S15 Appendix. Protocol for RNA extraction from neat sputum for RNAseq.**

|  | **Commercial**  **Buffers** | **Homemade**  **Buffer Substitutes** |
| --- | --- | --- |
| **Pre-lysis 1:** | PBS  0.5% n-lauryl sarcosine  1% 2-mercaptoethanol | 150mM NaCl  20mM Tris pH 7.4  0.5% n-lauryl sarcosine  1% 2-mercaptoethanol |
| **Pre-lysis 2*:** | RLT-plus | 4M Guanidine thiocyanate  1% Triton |
| **Pre-lysis 3*:** | RLT | 4M Guanidine thiocyanate |
| **Lysis 1*:** | RLT | 4M Guanidine thiocyanate |
| **Lysis 2*:** | RLT-plus | 4M Guanidine thiocyanate  1% Triton |
| **Wash 1*:** | RLT + 16.7% ISOH | 4M Guanidine thiocyanate  16.7% ISOH |
| **Wash 2^1^:** | 80% ETOH  10mM Tris pH 7 | 80% ETOH  10mM Tris pH 8 |
| **Elution:** | 10mM Tris pH 8 | 10mM Tris pH 8 |

**NOTE:**

- This protocol needs to performed inside a **biosafety level 3** laboratory and hood with appropriate biosafety protocols and personal protective equipment including **N95 mask**

- A list of all reagents and sources can be found at **S17 Appendix**

* These buffers contain guanidine thiocyanate which is INCOMPATIBLE with bleach

**Protocol**

1. Transfer up to 25mL of neat sputum into 50mL tube, screw lid tightly
   1. Rigorously vortex at maximum setting for up to 5min or until homogenized^2^
2. Let tube sit for 5min (to allow aerosols to settle), then pipette 333µL into 3 screwcap 1.7mL tubes^3^
   1. Use wide orifice 1mL tips
3. Pipette 666µL of **pre-lysis 1** into each of the three tubes
   1. Pipette up/down 10 times (use non-wide orifice 1mL tip), close lid tightly, vortex for 30 sec
4. Spin at max speed for 5min
5. Remove supernatant, resuspend the 3 pellets with 1mL of **pre-lysis 2** and combine into 1 tube
6. Spin at maximum speed for 5min, remove supernatant
7. Resuspend with 1mL **pre-lysis 3**, vortex
8. Spin at maximum speed for 5min, remove supernatant
9. Resuspend pellet with 500µL of **lysis 1** and transfer to a pre-chilled Beadbeater Zirconium tube
10. Bead beat for 30sec, chill for 1 min, repeat two more times
11. Spin max speed for 15sec
12. Add 400µL **lysis 2** into the tube, vortex, spin max speed 15sec
13. Transfer 750µL of supernatant into Omega DNA^4^ spin column
14. Spin max speed 30sec^5^
15. Add 145µL isopropanol to flow-through, mix by pipetting up/down and transfer 750µL to Omega RNA spin column^4^
16. Spin max speed 30 sec
17. Transfer column to new 2mL capless tube, pipette 500µL **Wash 1** to RNA column
18. Spin max speed 30sec
19. Transfer column to new 2mL capless tube, pipette 500µL **Wash 2** to RNA column
20. Spin max speed 1min
21. Transfer column to new 1.7mL tube, place in 60-65C warm plate for 5min to dry
22. Elute with 50µL **elution** buffer (pre-warm to 60-65C if you have many tubes)
23. Spin max speed for 1min
24. Add 5µL of 10X DNase buffer and 4µL of DNase (mix by tapping, DO NOT VORTEX), spin down
25. Incubate at 37C for 30min
26. Add 2µL 50mM EDTA, vortex vigorously
27. Incubate 65C for 10min
28. Spin down max speed, freeze at -80C

^1^ If eluting with H_2_0 (which is not recommended due to lower yield), use 80% ETOH 10mM Tris pH 8

^2^ Expectorated samples require more effort to homogenize, invert before vortexing

^3^ Samples can be frozen at -80C at this step, freeze in 1.2mL aliquots in screw cap tubes

^4^  Other DNA/RNA columns are compatible though the protocol has been optimized with Omega Biotek

^5^ DNA column can be saved if DNA purification is required (see **S16 Appendix**) or discarded if not

**S16 Appendix. Protocol for DNA extraction from neat sputum.**

|  | **Commercial**  **Buffers** | **Homemade**  **Buffer Substitutes** |
| --- | --- | --- |
| **Pre-lysis 1:** | PBS  0.5% n-lauryl sarcosine  1% 2-mercaptoethanol | 150mM NaCl  20mM Tris pH 7.4  0.5% n-lauryl sarcosine  1% 2-mercaptoethanol |
| **Pre-lysis 2*:** | RLT | 4M Guanidine thiocyanate |
| **Lysis 1*:** | RLT | 4M Guanidine thiocyanate |
| **Lysis 2*:** | RLT-plus | 4M Guanidine thiocyanate  1% Triton |
| **Wash 1*:** | RLT | 4M Guanidine thiocyanate |
| **Wash 2:** | 80% ETOH  10mM Tris pH 7 | 80% ETOH  10mM Tris pH 7 |
| **Elution:** | 10mM Tris pH 8 | 10mM Tris pH 8 |

**NOTE:**

- This protocol needs to performed inside a **biosafety level 3** laboratory and hood with appropriate biosafety protocols and personal protective equipment including **N95 mask**

- A list of all reagents and sources can be found at **S17 Appendix**

* These buffers contain guanidine thiocyanate which is INCOMPATIBLE with bleach

**Protocol**

1. Transfer up to 25mL of neat sputum into 50mL tube, screw lid tightly
   1. Rigorously vortex at maximum setting for up to 5min or until homogenized^2^
2. Let tube sit for 5min (to allow aerosols to settle), then pipette 333µL into 3 screwcap 1.7mL tubes^3^
   1. Use wide orifice 1mL tips
3. Pipette 666µL of **pre-lysis 1** into each of the three tubes
   1. Pipette up/down 10 times (use non-wide orifice 1mL tip), close lid tightly, vortex for 30 sec
4. Spin at max speed for 5min
5. Remove supernatant, resuspend the 3 pellets with 1mL of **pre-lysis 2** and combine into 1 tube
6. Spin at maximum speed for 5min, remove supernatant
7. Resuspend pellet with 500µL of **lysis 1** and transfer to a pre-chilled Beadbeater Zirconium tube
8. Bead beat for 30sec, chill for 1 min, repeat two more times
9. Spin max speed for 15sec
10. Add 400µL **lysis 2** into the tube, vortex, spin max speed 15sec
11. Transfer 750µL of supernatant into Omega DNA^4^ spin column
12. Spin max speed 30sec^5^
13. Transfer column to new 2mL capless tube, pipette 500µL **Wash 1** to DNA column
14. Spin max speed 30sec
15. Transfer column to new 2mL capless tube, pipette 500µL **Wash 2** to DNA column
16. Spin max speed 1min
17. Transfer column to new 1.7mL tube, place on 60-65C warm plate for 5min to dry
18. Elute with 50µL **elution** buffer (pre-warm to 60-65C if you have many tubes)
19. Spin max speed for 1min, freeze at -80C

^1^ If eluting with H_2_0 (which is not recommended due to lower yield), use 80% ETOH 10mM Tris pH 8

^2^ Expectorated samples require more effort to homogenize, invert before vortexing

^3^ Samples can be frozen at -80C at this step, freeze in 1.2mL aliquots in screw cap tubes

^4^  Other DNA columns are compatible though the protocol has been optimized with Omega Biotek Hibind columns

^5^ Flow through can be used for RNA extraction (see **S14-15 Appendix**) or discarded

**S17. Excel file outlining sources and cost of reagents needed for the RNA extraction and qRTPCR protocols.**

**S18. Primer and probe sequences used in this study.**

| Gene | Primer/ probe | Sequence (5’-3’) | qRTPCR Cycling conditions |
| --- | --- | --- | --- |
| 16S rRNA | F1 | ACG GG TGA GTA ACA CGT GGG TG | 50°C 20 minutes (RT)  95°C 15 minutes  95°C 30 seconds*  60°C 75 seconds  *Repeat 39 times |
|  | F2 | ACT GGG TCT AAT ACC GGA TAG GAC |  |
|  | F1.1 | CTG GGA AAC TGG GTC TAA TAC C |  |
|  | F1.2 | GAA ACT GGG TCT AAT ACC GGA TAG |  |
|  | R1 | AAG CTG ATA GGC CGC GGG CT |  |
|  | R4 | GGG CTC ATC CCA CAC CGC T |  |
|  | R1.2 | CTC ATC CCA CAC CGC TAA AGC |  |
|  | Probe | CY5- ACG GGA TGC ATG TCT TGT GGT GGA AAG CGC-BBQ |  |
| icl1 | F | GAC CCA GCA GCA CAT CCG CA |  |
|  | R | GTT CTT GGT GCG GTA GAA GC |  |
|  | Probe | FAM - CTC GGC TCG CGG CCG ATG TGG -QSY |  |
| IS6110 | F | CCG AGG CAG GCA TCC AAC |  |
|  | R | GAT CGT CTC GGC TAG TGC ATT |  |
|  | Probe | VIC- TCG GAA GCT CCT ATG AC - MGB |  |
| Human actin | F | ACC GAG CGC GGC TAC AG |  |
|  | R | CTT AAT GTC ACG CAC GAT TTC C |  |
|  | Probe | VIC-TTC ACC ACC ACG GCC GAG C -MGB |  |
| hspX | F | CAA GGA CGT CGA CAT TAT GGT C |  |
|  | R | CCG TCG AAG TCC TTC TGC TC |  |
|  | Probe | FAM-CAG CTG ACC ATC AAG GCC GAG CG-QSY |  |
| vapB10 | F | AAG CGG ACC AAC ATC TAC CTC |  |
|  | R | TGG TGA GGG CTC GGT TCA G |  |
|  | Probe | VIC-CAA GTT GGC CGC GCA AGA AGG TGT TTC-QSY |  |
| lldD2 | F | AAG GGT TCC GCG ACA TCG AG |  |
|  | R | GTT CCA GCC GGC GCA CAC |  |
|  | Probe | VIC-CCG ACA ATC CTG CGT GAC GTC ACC-QSY |  |
| carD | F | ACG TCG TTA CAA GGC GAA CCT C |  |
|  | R | CGC CAC AAA TCG CGC ACT AC |  |
|  | Probe | FAM-CAT CCG GCG ATG TGA ACA AGG TGG C -QSY |  |
| whiB1 | F | GGT CTG TCG TGA CGA GGA TC |  |
|  | R | TTA CAG ACC AGT TTC GCG TCA G |  |
|  | Probe | VIC-CTG TTC TTC CCG GTA GGA AAC AGT GGT-QSY |  |
| tatA | F | GCG GAA GCG TCT ATC GAG AC |  |
|  | R | CTT GAC CAG ACG CCG CTG AG |  |
|  | Probe | FAM-CCG GTG CAA TCG CAG CGT GTC GA-QSY |  |
| Rv1738 | F | CGA CCA GTC GGA TCA CGT G |  |
|  | R | ACC AAT TCC TTT TCC CGC CAA C |  |
|  | Probe | CGA CGA ACA CGA AGG ATT GAC TCG GG |  |
| arsC | F | GAT CAT GAT GTA TCC GGT GCT G |  |
|  | R | GAG TAG CAG CTT GCG GTC AC |  |
|  | Probe | FAM-CTA CGA CCG CCT CGA CAC CGT CA-QSY |  |
| lpqX | F | AGC TGA CCG GCA AGG CTAC |  |
|  | R | GAA CCG ACG TTC CCC TTC AG |  |
|  | Probe | VIC-CTT ATC CTC GTC GAA ACC AGT TGC GG-QSY |  |
| sigA | F | GTG ACC CGG GAA CGC ATC |  |
|  | R | CGG CTC GGA TGG CGC AAC |  |
|  | Probe | CY5- CGC CAG ATC GAA TCC AAG ACT ATG TCG A-BBQ |  |

Cy5, cyanine; BBQ, BlackBerry Quencher; VIC (life Technologies); MGB, minor-groove binder; qPCR, quantitative polymerase chain reaction; RT, reverse-transcription

**S19. Excel file showing culture and qRTPCR results for all patient sputa samples examined in this study.**
